# Immunoadsorption Versus Sham Treatment for Post-COVID Syndrome: A Randomised Sham-Controlled Crossover Trial

**DOI:** 10.1016/j.lanepe.2026.101744

**Published:** 2026-06-11

**Authors:** Marco Stortz, Andreas Kommer, Myriam Meineck, Simone Cosima Boedecker-Lips, Paul Claßen, Vanessa Tomalla, Felix Rausch, Livia Sophie Lang, Philipp S. Wild, Irene Schmidtmann, Arndt Weinmann, Daniel Kraus, Julia Weinmann-Menke

**Affiliations:** a I. Department of Medicine, University Medical Centre of Johannes Gutenberg University Mainz, Mainz, Germany; b Preventive Cardiology and Preventive Medicine, Department of Cardiology, University Medical Centre, Johannes Gutenberg University, Mainz, Germany; c Department of Neurology, University Medical Centre, Johannes Gutenberg University, Mainz, Germany; d Clinical Epidemiology and Systems Medicine, Centre for Thrombosis and Haemostasis, University Medical Centre, Johannes Gutenberg University, Mainz, Germany; e German Centre for Cardiovascular Research (DZHK), Partner Site Rhine-Main, Mainz, Germany; f Systems Medicine, Institute of Molecular Biology (IMB), Mainz, Germany; g Institute for Medical Biometry, Epidemiology and Informatics, University Medical Centre of the Johannes Gutenberg University Mainz, Mainz, Germany; h Centre of Immunotherapy (FZI), Johannes Gutenberg University, Mainz, Germany

**Keywords:** SARS-CoV-2, Autoimmunity, Autoinflammation, Sequelae, Immunoadsorption, Trial

## Abstract

**Background:**

Post-Covid syndrome is a debilitating condition which may be caused and/or aggravated by autoantibodies. The current study aimed to determine whether autoantibody depletion by immunoadsorption is effective to reduce the symptom burden of patients with post-Covid syndrome.

**Methods:**

IAMPOCO was a randomised, patient-blinded, sham-controlled crossover trial of immunoadsorption with tryptophan adsorbers versus sham treatment in patients with post-Covid syndrome at a tertiary academic care centre. The primary outcome was the difference in change in symptom severity before and after the respective therapies. Secondary outcomes included treatment safety, prevalence of autoantibodies against G protein-coupled receptors (adrenergic and muscarinergic receptors), and the influence of treatment on autoantibody levels.

**Findings:**

40 patients with post-Covid syndrome and a symptom severity of at least 2 on the Post-Covid-19 Functional Scale were included and randomised to a treatment sequence. There was no difference in change in symptom severity between immunoadsorption and sham; odds ratio in Post-Covid-19 Functional Scale, OR = 1.17 (95% CI, 0.41–3.36; p = 0.771), mean difference in MFI-20 2.4 (95% CI, −3.7–8.5; p = 0.437), in CFS 0.09 (95% CI, −4.5 to 4.7; p = 0.970), in Bell-Scale −2.6 (95% CI, −6.9 to 1.8; p = 0.246), in MoCA score −0.01 (95% CI, −1.2 to 1.1, p = 0.993) and in Handgrip-strength deviation from individual normal value 1.3 (−0.83 to 3.5, p = 0.234). 34 adverse events occurred, 10 during or after sham treatment and 24 during or after immunoadsorption. Autoantibodies against G protein-coupled receptors were depleted by immunoadsorption but not sham treatment.

**Interpretation:**

Immunoadsorption was not effective in reducing symptom burden in post-Covid syndrome.

**Funding:**

Ministry of Health, state of Rhineland-Palatinate; Mainz University Medical Centre; DIAMED Medical Technology. Trial Registration: ClinicalTrials.gov, number NCT05841498.

**Research in context**

**Evidence before this study**

Despite the large number of people affected worldwide since the start of the SARS-CoV-2 pandemic, there is no specific treatment for post-COVID syndrome (PCS). The pathophysiology of the syndrome remains poorly understood but appears to involve at least four distinct mechanisms: virus persistence and associated tissue damage; an excessive immune response to the initial viral infection with persistent inflammation and autoimmunity; endothelial dysfunction and immune-related thrombosis; and psychological or psychosomatic factors. In order to target the autoimmunity aspect, patients are being treated with immunoadsorption, but evidence from randomised controlled trials is lacking to show its effectiveness and safety.

Planning a randomised crossover trial of immunoadsorption for the treatment of PCS, we conducted a systematic search of the PubMed database in February 2023 using the search terms “post COVID,” “long COVID,” “immunoadsorption,” and “extracorporeal therapy” to find evidence of immunoadsorption in post-COVID syndrome without any year or language restriction. Only one case series was found. A search on Clinicaltrials.gov in February 2023 revealed that another randomised controlled trial was planned in a parallel-group design in patients with myalgic encephalomyelitis/chronic fatigue syndrome (ME/CFS). This trial should also include patients with PCS. During later searches on ClinicalTrials.gov, another randomised controlled trial with a parallel-group design was found, which was registered in July 2023.

To the best of our knowledge, no results from either study had been published at the time of manuscript preparation. While our study was underway, an additional randomised controlled trial was published that investigated the effect of plasma exchange on symptom severity in PCS.

**Added value of this study**

This is the first randomised controlled trial of immunoadsorption in PCS. Compared with sham treatment, immunoadsorption with tryptophan adsorbers did not provide a clinical benefit. Adverse events occurred more often with immunoadsorption than with sham treatment.

**Implications of all the available evidence**

The pathophysiology of PCS is complex. While autoimmunity may play a role in PCS, immunoadsorption with tryptophan adsorbers is unlikely to provide a clinically relevant therapeutic effect.

## Introduction

With over 777 million infections reported so far, severe acute respiratory syndrome coronavirus 2 (SARS-CoV-2) poses extreme challenges to healthcare systems worldwide.^1^ Many patients experience ongoing symptoms even after recovering from acute infection, including those with mild cases.^1^ This condition was named, among other terms, Post-COVID Syndrome (PCS). It is defined as symptoms that occur 3 months after an initial confirmed or suspected infection with SARS-CoV-2, persist for at least 2 months, and cannot be otherwise explained.^2^^,^^3^ It is estimated that among non-hospitalised subjects with SARS-CoV-2 infection, up to 10% develop PCS.^2^^,^^4^ Many individuals suffering from PCS can no longer work or can only do so in a limited capacity.^5^ Thus, besides the reduced quality of life for those affected, PCS represents a significant economic issue.^5^

PCS is a debilitating, albeit heterogeneous condition.^3^ Subjects often suffer from severe fatigue, post-exertional malaise, and other symptoms that prevent them from performing activities of daily living.^3^ Risk factors for developing PCS are female sex, high BMI, older age, smoking status, and severe Covid-19 disease, while vaccinated subjects appear to have a lower risk.^6^ Many tools, such as the Montral Cognitive Assessment (MoCA) or the Post-Covid Functional Scale (PCFS) and others, have been used in clinical research of PCS,^7^ and many approaches to treatment have been explored, but to date, optimal therapeutic management is elusive,^8^ and the pathophysiology of PCS remain poorly understood.

Autoimmunity is one of the proposed mechanisms in the pathogenesis of PCS.^9^ Various autoantibodies (Aabs) have been identified in patients with PCS, including antibodies against G-protein coupled receptors.^10^ Autoantibodies can also be found in healthy individuals,^11^ and pathophysiological significance in PCS has not been definitively established.^12^^,^^13^ Nonetheless, for lack of therapeutic alternatives, many desperate patients that are tested positively for the presence of autoantibodies seek extracorporeal therapies such as immunoadsorption (IA),^14^ even though there is currently no evidence beyond case reports or small case series to support benefits of such therapies.^15^^,^^16^

We initiated a pragmatic, single-blinded, randomised sham-controlled crossover intervention study, the Immunoadsorption study Mainz in adults with post-COVID syndrome (IAMPOCO), to test the hypothesis that immunoadsorption has a clinically relevant impact on the severity of PCS symptoms compared to sham therapy.

## Methods

### Study design and procedure

IAMPOCO is a patient-blinded, randomised, sham-controlled, cross-over study. The design and study protocol of IAMPOCO have been published previously.^17^ The study was conducted from May 2023 to October 2024 at the Department for Nephrology of the University Medical Centre Mainz, Germany.

All study subjects were initially assigned to a treatment sequence by randomisation, with either IA before sham treatment or vice versa. Visits were conducted before each treatment cycle, likewise two weeks after the end of each treatment cycle and 6–8 weeks after the end of the second treatment cycle. Between the two treatment cycles, there was a washout phase of eight weeks. At each visit the severity of symptoms was recorded using questionnaires, cognitive impairments were evaluated using Montreal cognitive assessment (MoCA) and grip strength was measured using a hand dynamometer. Furthermore, at each visit concentrations of immunoglobulins, complement-factors, a full blood count, the concentration of C-reactive protein, and herpes virus nucleic acid counts were obtained. Serum concentrations of autoantibodies (Aabs) against G protein-coupled receptors (GPCR) were measured before the first and after the last session of each treatment cycle. SARS-CoV-2 infection history and SARS-CoV-2 vaccination status were obtained from the patients’ medical history.

### Ethical approval

The study protocol was reviewed and approved by the Ethics Committee of the Regional Medical Association of Rhineland-Palatinate (no. 2022–16804). Written informed consent was given by all participants before any trial procedure. The trial is registered at ClinicalTrials.gov, number NCT05841498, 25 April 2023.

### Patients

Eligible patients were at least 18 years old, met the WHO^3^ definition of PCS and exhibited a symptom severity of category 2 or higher on the Post-COVID Functional Scale.^18^ To ensure that the symptoms were not attributable to another condition, participants were recruited from a cohort that either had previously taken part in a study on the prevalence of PCS, the Gutenberg Post-COVID study, or had undergone thorough examination for the presence of competing causes of the symptoms by a specialised PCS clinic or outpatient department.

Exclusion criteria were a previous or current psychiatric diagnosis, previous or current antibody-mediated autoimmune disease, pregnancy, allergy against any materials used for IA and other contraindications for IA.

### Patient and public involvement

Patients and the general public were not formally involved in the design, execution, and analysis of this study.

### Randomisation and masking

Randomisation to a treatment sequence was applied before the first treatment cycle by using an unrestricted random number generator by the principal investigator or the deputy of the principal investigator at the time of enrolment of every participant; given the cross-over design of the study, there was no allocation concealment. Patients were blinded to their assignment: For both IA and sham procedure, the devices were placed behind a portable wall and covered with a curtain so that they were not visible to the patient. However, since the setting-up of the machines differs depending on the procedure, it was not possible to blind the supervising nurse as well.

### Procedures

IA and sham treatment were conducted with the Plasauto Sigma® extracorporeal therapy system (Asahi Kasei, Tokyo, Japan) in combination either with the Immusorba® TR-350 adsorber (DIAMED medical technology, Cologne, Germany) or without adsorber on days 1, 2, 3, 5 and 7. During the first session 2 L of the participant's plasma were processed, during each other session 2.5 L, according to the manufacturer's recommendations. This regimen has been established by studies with neurological autoimmune diseases.^19^ Anticoagulation was performed with citrate in combination with unfractionated heparin. To ensure that the sham therapy was indistinguishable from IA for the subjects, the location, duration of therapy and blood samples were the same as for IA.

### Outcomes

The primary outcome was the impact of IA on PCS symptom severity measured by changes in:

- Post-COVID Functional Scale (PCFS) ranging from 0 to 4^18^
- Chalder Fatigue Scale (CFS) ranging from 0 to 33^20^
- Bell Score ranging from 0 to 100^21^
- Multidimensional Fatigue Inventory (MFI-20) ranging from 20 to 100^20^
- Montral Cognitive Assessment (MoCA) ranging from 0 to 30^22^
- Hand-grip strength^23^ before and two weeks post IA and sham apheresis.

Secondary outcomes were the safety of IA in contrast to the sham procedure, assessment of G-protein-coupled receptor autoantibody prevalence and alterations in their concentration under the respective therapy.

### GPCR autoantibodies

Aabs were analysed with assay kits by CellTrend (Luckenwalde, Germany). Samples were pre-diluted 1:100. The qualitative assessment of the results (positive, negative, or “higher risk”, which is a term used by the manufacturer to indicate an intermediate result) was based on the reference values provided by the manufacturer, where reference values were specified. Values above the upper reference range (>80 U/L) were censored at 80 U/L.

### Statistical analysis

We determined that the enrolment of 38 patients would provide the trial with 90% power, to detect a clinically relevant improvement of the MoCA score of at least 2 points with IA. To account for potential dropout, 40 participants were to be included.

The power was calculated for a parallel group design and a two-sample test. The cross-over design—which is more effective than a simple parallel group design—was not accounted for. This was in order to still have sufficient power in case too many drop-outs would require a restriction of analysis to the first cycle. Thus, the actual power is higher than what the sample size calculation for the parallel group design yields.

For all quantitative primary and secondary endpoints, a mixed linear model with treatment and period as fixed effects and patient as a random effect was used to evaluate the significance of the influence of treatment and period on the changes under treatment. Marginal estimates and 95% confidence intervals for the effects of treatment are reported, as well as p values for the main effects of treatment. Additionally, it was assessed whether a carry-over effect must be assumed. A generalised mixed linear model was applied for the PCFS as an ordinal primary endpoint, analogous to the mixed linear model to the quantitative primary endpoints, with treatment and period as fixed effects and patient as a random effect. The Bonferroni-Holm method was used to adjust for multiple testing.

In addition to the mixed linear model for the effect of the respective therapy on autoantibody concentration, the absolute and relative frequencies of patients with detectable antibodies were determined, including mean and standard deviation at baseline and stratified by treatment (verum/placebo).

Adverse events were collected for up to 6 weeks after the end of each cycle and were stratified by period and treatment. From this, absolute and relative frequencies of adverse events of the respective severity grades under the treatment modalities were calculated.

One serum sample was lost in transit. There is no discernible association with any independent or dependent variable in our study. Therefore we consider it to be completely missing at random, not introducing any bias into the analysis.

### Role of the funding source

The trial was funded by the University Medical Centre Mainz and the Ministry of Health of the State Rhineland–Palatine. Materials were provided by DIAMED Medical Technology, Cologne, Germany. The funders had no role in the design of the study, data collection, analysis, interpretation, manuscript writing, or the decision to submit for publication.

## Results

### Patients

**Fig. 1:**
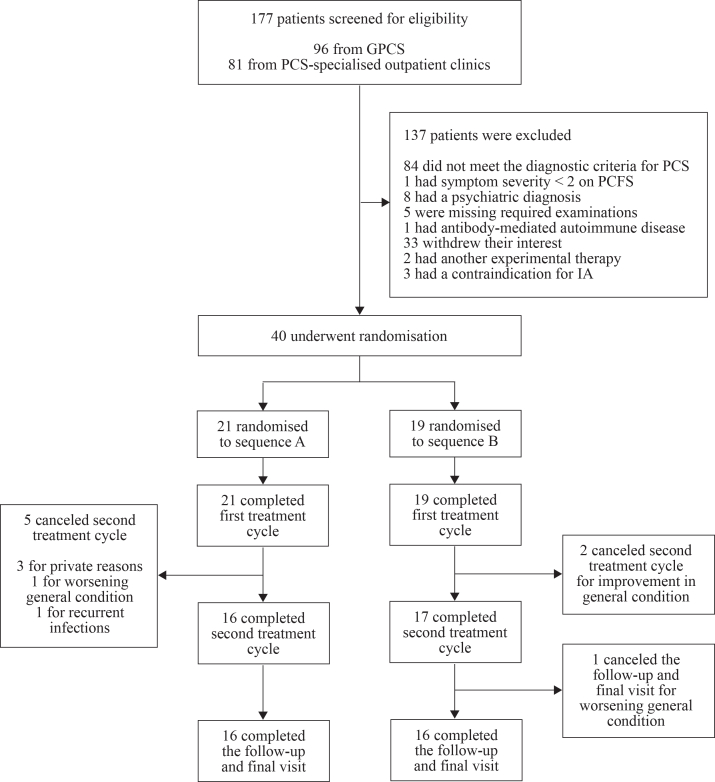
Enrolment, randomisation, treatment sequence and follow-up. GPCS, Gutenberg Post-Covid Study; IA, immunoadsorption; PCFS, Post-Covid Functional Scale; PCS, post-Covid syndrome.

The detailed recruitment and study flow is shown in Fig. 1. As listed in Table 1 40 patients, with a mean age of 42 years, 72.5% female, 70% currently unfit for work and a median disease duration of 21.6 months were enrolled and randomised to a treatment sequence (21 to IA first and 19 to sham first). All subjects were Caucasian. All patients underwent at least the first treatment cycle, and 34 patients underwent the second (16 in the “IA first” group and 18 in the “sham first” group). Most patients were severely affected or care-dependent due to PCS with 32.5% and 37.5% of patients having a score of 3 or 4 on the PCFS, respectively. Fatigue was also severe with a mean score of 78.7 ± 8.7 points on the MFI-20. The deviation of grip strength from age-, height- and gender-adjusted normal values was 11.4 ± 11 kg. The exploratory neurocognitive testing was mostly normal, with a mean of 26.5 ± 2.8 points on the MoCA.

**Table 1:** Baseline characteristics of patients.

Characteristic	Treatment sequence		Total N = 40
	Sequence IA-Sham N = 21	Sequence Sham-IA N = 19	
Age [years]	39.1 (15.6)	44.8 (11.0)	41.8 (13.7)
Sex			
F	15 (71.4)	14 (73.7)	29 (72.5)
M	6 (28.6)	5 (26.3)	11 (27.5)
Body mass index [kg/m^2^]	24.5 (4.5)	24.1 (4.1)	24.3 (4.3)
Duration of disease [months]	18.5 (9.3)	24.9 (11.0)	21.6 (10.5)
Course SARS-COV2 infection			
Asymptomatic	0 (0.0)	1 (5.3)	1 (2.5)
Hospital general condition	0 (0.0)	2 (10.5)	2 (5.0)
Hospital oxygen	1 (4.8)	0 (0.0)	1 (2.5)
Mild symptoms	13 (61.9)	10 (52.6)	23 (57.5)
Severe symptoms home	7 (33.3)	6 (31.6)	13 (32.5)
Number of infections (Infection after PCS began/total number of infections), n			
1/1	19 (90.5)	10 (52.6)	29 (72.5)
1/2	1 (4.8)	7 (36.8)	8 (20.0)
1/3	0 (0.0)	1 (5.3)	1 (2.5)
2/2	0 (0.0)	1 (5.3)	1 (2.5)
3/5	1 (4.8)	0 (0.0)	1 (2.5)
Patients unfit to work	14 (66.7)	14 (73.7)	28 (70.0)
Vaccination against SARS-COV2			
0	2 (9.5)	0 (0.0)	2 (5.0)
1	0 (0.0)	2 (10.5)	2 (5.0)
2	0 (0.0)	5 (26.3)	5 (12.5)
3	17 (81.0)	9 (47.4)	26 (65.0)
4	2 (9.5)	3 (15.8)	5 (12.5)
Concentration of complement factors			
C1q [g/l]	0.2 (0.03)	0.2 (0.04)	0.19 (0.25)
C4 [g/l]	0.22 (0.06)	0.21 (0.07)	0.22 (0.06)
C3c [g/l]	1.11 (0.21)	1.14 (0.2)	1.07 (0.21)
Autoantibodies			
Muscarinic acetylcholine receptors, Subtype 1			
Detectable^1^	21 (100.0)	19 (100.0)	40 (100.0)
Muscarinic acetylcholine receptors, Subtype 2			
Neg	15 (75.0)	18 (100.0)	33 (86.8)
Pos	5 (25.0)	0 (0.0)	5 (13.2)
Muscarinic acetylcholine receptors, Subtype 3			
Neg	1 (5.0)	2 (10.5)	3 (7.7)
Intermediate^b^	7 (35.0)	11 (57.9)	18 (46.2)
Pos	12 (60.0)	6 (31.6)	18 (46.2)
Muscarinic acetylcholine receptors, Subtype 4			
Neg	10 (50.0)	12 (63.2)	22 (56.4)
Pos	10 (50.0)	7 (36.8)	17 (43.6)
Muscarinic acetylcholine receptors, Subtype 5			
Detectable^a^	21 (100.0)	19 (100.0)	40 (100.0)
Alfa adrenoreceptors, Subtype 1			
Neg	4 (20.0)	3 (15.8)	7 (17.9)
Intermediate^b^	3 (15.0)	5 (26.3)	8 (20.5)
Pos	13 (65.0)	11 (57.9)	24 (61.5)
Alfa adrenoreceptors, Subtype 2			
Neg	1 (5.0)	2 (10.5)	3 (7.7)
Pos	19 (95.0)	17 (89.5)	36 (92.3)
Beta adrenoreceptors, Subtype 1			
Neg	11 (55.0)	12 (63.2)	23 (59.0)
Pos	9 (45.0)	7 (36.8)	16 (41.0)
Beta adrenoreceptors, Subtype 2			
Neg	5 (25.0)	8 (42.1)	13 (33.3)
Intermediate^b^	3 (15.0)	5 (26.3)	8 (20.5)
Pos	12 (60.0)	6 (31.6)	18 (46.2)
Presence of fatigue	20 (95.2)	16 (84.2)	36 (90.0)
Lack of concentration	11 (52.4)	11 (57.9)	22 (55.0)
Presence of myalgia	10 (47.6)	5 (26.3)	15 (37.5)
Presence of arthralgia	2 (9.5)	1 (5.3)	3 (7.5)
Presence of tachycardia	2 (9.5)	2 (10.5)	4 (10.0)
Bedridden Patients	0 (0.0)	1 (5.3)	1 (2.5)
Presence of pain	4 (19.0)	4 (21.1)	8 (20.0)
Hand-Grip-strength deviation from the age-, gender-, and size-dependent standard value [kg]	−12.1 (12.7)	−10.6 (9.0)	−11.4 (11.0)
Post-COVID-19-functional scale (PCFS)			
1	0 (0.0)	1 (5.3)	1 (2.5)
2	9 (42.9)	2 (10.5)	11 (27.5)
3	8 (38.1)	5 (26.3)	13 (32.5)
4	4 (19.0)	11 (57.9)	15 (37.5)
Post COVID score [points]	38.6 (7.8)	40.2 (7.7)	39.3 (7.7)
Multidimensional Fatigue inventory (MFI-20) [points]	79.2 (9.1)	78.2 (8.5)	78.7 (8.7)
Bell score [points]	38.1 (17.2)	31.6 (17.7)	35.0 (17.5)
Chalder fatigue scale [points]	26.1 (4.8)	28.1 (3.9)	27.1 (4.5)
MRC-Scale			
0	4 (19.0)	2 (10.5)	6 (15.0)
1	5 (23.8)	5 (26.3)	10 (25.0)
2	7 (33.3)	8 (42.1)	15 (37.5)
3	2 (9.5)	2 (10.5)	4 (10.0)
4	3 (14.3)	2 (10.5)	5 (12.5)
Montreal cognitive assessment (MOCA) [points]	25.9 (3.3)	27.1 (2.1)	26.5 (2.8)
Pain intensity in numerous analog scale	3.5 (2.1)	2.8 (2.3)	3.2 (2.2)

All categorical variables are given as numbers (%), all numerical variables as mean values (standard deviation). Percentages always refer to the number of patients with available data. The baseline values were available for all patients; only the post-COVID score and the concentration of antibodies against the muscarinic ACh receptor subtype 2 were missing for one patient in each group, and the concentration of antibodies against the other G protein-coupled receptors was missing for one patient in the IA-first group.

a No reference values specified by the test manufacturer.

b “Intermediate” denotes non-negative results that are not clearly positive either; the manufacturer of the assay uses the term “higher risk” for these test results in their documentation.

### Symptom severity

**Fig. 2:**
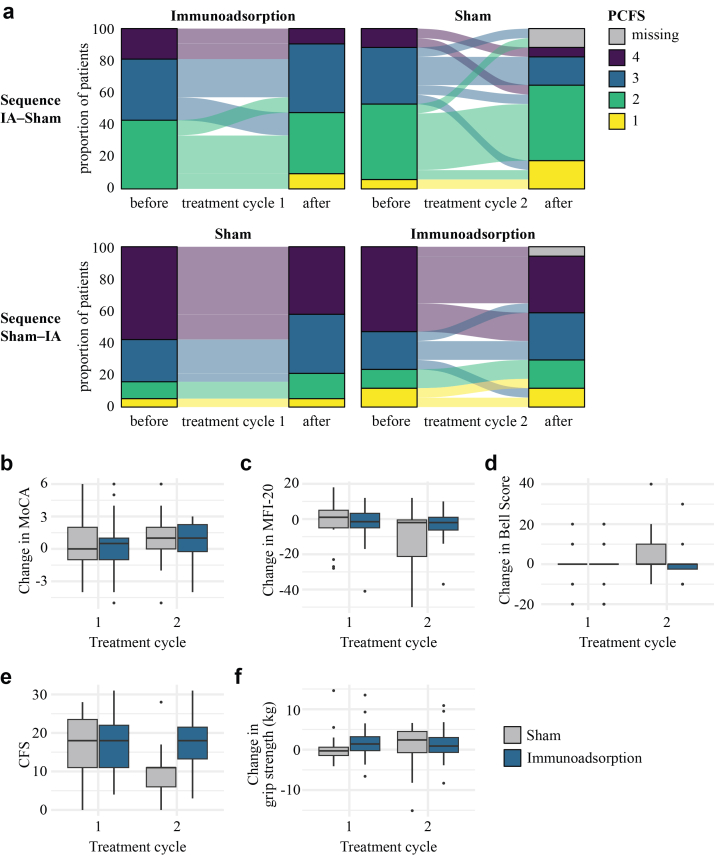
Changes in symptom severity for each test according to the respective therapy. (a) Distribution and changes of Post-Covid Functional Scale (PCFS) values before and after each treatment cycle. 1: negligible functional limitations, 2: slight functional limitations, 3: moderate functional limitations, 4: severe functional limitations. The stacked bars visualise the proportions of subjects with PCFS values before (left bar) and after (right bar) a treatment cycle. Light-shaded areas in between visualise individual changes, with a flowing appearance only if there is crossover in PCFS categories. (b) Change in Montral Cognitive Assessment Scale (MoCA); (c) change in Multidimensional Fatigue Inventory (MFI)-20 score; (d) change in Bell Score; (e) Chalder Fatigue Scale (CFS); (f) change in grip strength. Horizontal bars in boxes denote median values; upper and lower ends of boxes denote the first and third quartiles; whiskers denote the range of data without outliers; dots denote outliers.

No clinically relevant difference was found between IA and sham treatment for changes in either post-COVID symptom severity related to PCFS or the severity of fatigue based on MFI-20, CFS and Bell Scale before and after the respective treatment (Table 2, Fig. 2, Supplementary Figure S1). The odds ratio (OR) for an increase (clinical worsening) in PCFS score was 1.17 (95% confidence interval [CI], 0.41–3.36; p = 0.771; Table 2). The mean difference of change in MFI-20 between immunoadsorption and sham was 2.4 (95% CI, −3.7 to 8.5; p = 0.437; Table 2). The mean difference of change in CFS was 0.09 (95% CI, −4.5 to 4.7; p = 0.970; Table 2). The mean difference in change in Bell was −2.6 (95% CI, −6.9 to 1.8; p = 0.246; Table 2).

**Table 2:** Changes in symptom severity for each assessment method due to the respective therapy and the results of the mixed model analyses for comparison of the changes.

	Immunoadsorption	Sham treatment	Mean difference of change between Immunoadsorption and Sham-treatment, beta (95% confidence interval)^b^	p for treatment effect in linear mixed model analysis
Change in PCFS category	−2: 1 (2.7)	−2: 2 (5.9)	1.17 (0.41–3.36)	0.771
	−1: 10 (27)	−1: 6 (17.6)		
	0: 22 (59.5)	0: 26 (76.5)		
	1: 4 (10.8)	1: 0 (0)		
Changes in MFI-20 score, mean (SD)	−3.1 (11.1)	−5.6 (15.1)	2.4 (−3.7–8.5)	0.437
CFS^a^, mean (SD)	17.2 (7.6)	13.8 (7.9)	0.09 (−4.5–4.7)	0.970
Change in Bell-Scale, mean (SD)	0.0 (10.3)	2.6 (10.1)	−2.6 (−6.9–1.8)	0.246
Change in MoCA score, mean (SD)	0.6 (2.4)	0.6 (2.5)	−0.01 (−1.2–1.1)	0.993
Change Handgrip strength deviation from individual normal value, mean (SD) [kg]	1.7 (4.6)	0.4 (5.0)	1.3 (−0.83–3.5)	0.234

a Since the CFS is measured in relation to a reference period, the change in the CFS score is not indicated, but rather the value after the respective therapy cycle.

b For PCFS it is the odds ratio between adjacent categories in a proportional odds model. −2 and −1 were combined into one category (decrease in PCFS).

Also, the differences in grip strength before and after treatment did not differ between the treatment modalities, nor did cognitive impairment, which was assessed using MoCA. The mean difference in change in grip strength was 1.3 (95% CI, −0.83 to 3.5; p = 0.234; Table 2). The mean difference in change in MoCA was −0.01 (95% CI, −1.2 to 1.1; p = 0.993; Table 2).

Because PCS may respond differently to IA in women and in men, the analyses were repeated for the individual sexes. In women, IA did not significantly affect any of the outcomes. In the 11 men that were included in the study, IA was associated with a worsening in PCFS (odds ratio, 2.12; p < 0.001) and MFI-20 (beta, 8.4; 95% confidence interval, 1–16; p = 0.042), and no significant nor meaningful changes in the other outcomes.

Furthermore, to account for potential imbalance in the randomised groups at baseline, additional exploratory analyses were performed that included age, duration of post-Covid syndrome, course of the index infection, number of infections, and vaccination status as covariates (Supplementary Tables S1–S13). With regard to the PCFS outcome, there was a statistically significant association of a severe course of the index infection with immunoadsorption; however, when an interaction term was fitted, the odds ratio for subjects with a *mild* index infection having a *worse* PCFS score after immunoadsorption was statistically significant (OR 6.63; 95% CI, 1.16–38; p = 0.034; Supplementary Table S3). For all other outcomes, the mixed-model covariate analyses did not reveal further insights.

### Safety

34 adverse events occurred in 22 patients, 10 of which occurred during or after the sham treatment and 24 during or after IA (Supplementary Table S14). Of these adverse events, 23 (14 during or after IA) were classified as mild and 11 (10 during or after IA) as moderate. The most common mild adverse events during or after IA were clotting of the adsorber or the tube system (3), mild infections (2), and central venous puncture difficulties (2). During or after the sham treatment, iron deficiency (3) and herpes virus reactivation (2) were the most common adverse events.

The most serious and most frequent moderate complications were jugular vein thromboses associated with central venous catheters for extracorporeal therapy, all of which occurred after the treatment cycle with IA. One moderate adverse event occurred in the form of syncope after sham treatment.

### Treatment efficacy and autoantibodies against G protein-coupled receptors

Immunoadsorption, but not sham treatment, resulted in a strong decline in serum IgG levels (Supplementary Figure S2).

39 of 40 patients had at least one detectable GPCR autoantibody. Baseline concentrations were missing for one patient. IA but not sham treatment effectively reduced autoantibody levels (Table 3, Supplementary Figure S3, Supplementary Table S15).

**Table 3:** Concentrations of each GPCR autoantibody at baseline and changes in concentration (concentration after treatment cycle—concentration before treatment cycle) due to the respective treatment modality and p value of mixed linear models for difference in concentration change between IA and sham treatment.

	Mean concentration initial [U/l] (SD), n = 39	Mean change in concentration by immunoadsorption [U/l] (SD), n = 35	Mean change in concentration by sham [U/l] (SD), n = 35	p value for treatment effect, linear mixed model for difference between immunoadsorption and sham
Anti-M1-Aab	6.1 (6.3)	−4.8 (5.2)	0.7 (5.9)	<0.001
Anti-M2-Aab	8.0 (12.2)	−3.2 (2.1)	−3.3 (11.3)	0.403
Anti-M3-Aab	25.7 (30.3)	−16.0 (22.7)	−0.8 (6.9)	<0.001
Anti-M4-Aab	22.0 (24.6)	−13.2 (16.2)	−2.8 (7.9)	<0.001
Anti-M5-Aab	11.6 (4.3)	−4.7 (3.0)	−0.7 (2.2)	<0.001
Anti-α1-Aab	27.8 (29.6)	−18.1 (25.1)	−1.0 (14.0)	<0.001
Anti-α2-Aab	44.1 (24.5)	−20.6 (21.3)	−3.5 (8.3)	<0.001
Anti-β1-Aab	25.0 (25.5)	−14.9 (16.3)	−1.1 (5.9)	<0.001
Anti-β2-Aab	26.0 (29.1)	−14.2 (20.2)	−1.7 (3.9)	<0.001

## Discussion

This study is the first randomised, single-blinded, sham-controlled trial to investigate the effect of IA on the severity of symptoms in patients with PCS according to the WHO definition and found no effect. Although IA reduced GPCR autoantibody levels in patients with detectable levels at baseline, symptom severity did not change compared to sham treatment, which had no effect on antibody levels. In addition, more adverse events occurred during or after IA in comparison to sham treatment.

IA as well as the related technique therapeutic plasma exchange are used to treat various autoantibody-mediated autoimmune diseases in addition to pharmacological treatments.^24^ In therapeutic plasma exchange, the patient's plasma is separated from the cellular blood components, discarded and substituted with plasma substitute solutions or fresh plasma, while IA primarily removes specific immunoglobulins from the patient's plasma adsorptively.^24^ Numerous studies have shown the therapeutic benefit of these procedures in various autoimmune diseases.^24^ Due to the suspected pathogenetic significance of autoantibodies against GPCRs in PCS, these methods have also been applied to this syndrome. The use of these procedures was reasoned by the similarity of PCS symptoms to those of myalgic encephalomyelitis/chronic fatigue syndrome (ME/CFS), in which a pathogenetic role of these autoantibodies has also been postulated, and different therapeutic apheresis procedures have been used in studies.^25^ Extracorporeal treatments were also used in light of possible microclot formations in the context of a persistent inflammatory response, although a Cochrane analysis found no therapeutic benefit here either.^26^ Case series without blinding and without control intervention had suggested a positive effect of IA with regard to an improvement in symptom severity in PCS with the clinical features of ME/CFS.^27^, ^28^, ^29^ In contrast, our study showed no effect on symptom severity compared to a sham treatment, which is in line with the results of another parallel-group randomised controlled trial that examined the effect of therapeutic plasma exchange on symptom severity in PCS with an even greater symptom improvement following sham therapy.^30^ While the other randomised study was primarily designed to look at differences in the safety of the procedures, the effects of treatment on symptom severity were only secondary endpoints and no autoantibody concentrations or other therapeutic goals of extracorporeal therapy were measured.^30^ Differences in the results between RCTs and case series can be explained by the therapy-independent context effect, which, depending on the intervention, can account for up to 54% of the observed effect and is not caused by the therapeutic intervention itself.^31^ Given the extracorporeal nature of the therapies, at least a placebo effect would have been expected in our study.^31^ Indeed, several patients in our study reported improvements in various symptom scores after sham treatment, consistent with a placebo effect; however, similar patterns of improvement and worsening were also observed after IA. The fact that placebo effects may occur with extracorporeal therapies, too, underscores the necessity to perform sham-controlled trials. For example, a recent study of repeat immunoadsorption in PCS patients did not include a control group, and selected only those patients for a second treatment cycle that reported an improvement after the initial treatment, thereby risking exaggerating the observed effect.^29^ Another recent trial with PCS patients that reported a placebo-controlled double-blind design compared plasma exchange (active comparator) with saline infusions (placebo), but an infusion may not be an adequate sham procedure for plasma exchange and achieving blinding appears impossible.^30^ The crossover and sham-controlled design is a major strength of the current trial that sets it apart from all other studies and case series published to date.

ME/CFS and post-exertional malaise (PEM) presentations were not a focus of our study due to the prior experience at our centre with patient referrals that included subjects with other manifestations as well.

While clinical outcomes do not definitively prove or disprove pathophysiological mechanisms, the efficacy or lack of efficacy of treatments targeting specific immunological processes can at least provide clues to the underlying pathogenic mechanisms. The pathogenesis of PCS is still unclear.^9^ Four mechanisms are currently considered to be the main explanatory approaches: viral persistence and associated tissue damage, an excessive immune response with persistent inflammation and autoimmunity, psychological factors respectively somatic symptom disorders, as well as endothelial inflammation and immune thrombosis.^9^^,^^32^ One mechanism of autoimmunity is the formation of autoantibodies directed against various tissues or receptors. Indeed, a number of autoantibodies have been associated with PCS to date. In our study, IA depleted GPCR autoantibodies in all patients who had elevated levels of autoantibodies at baseline, whereas these levels were not affected by sham treatment. Nevertheless, there was no difference in the primary endpoints between the two treatment modalities, so that the pathogenetic significance or at least the clinical relevance of these autoantibodies seems questionable. Further studies on the pathogenetic mechanisms behind PCS have been published since IAMPOCO was conceived, showing inconsistent results regarding the relevance of autoantibodies in PCS. None showed an association of PCS with GPCR autoantibodies.^33^^,^^34^ Importantly, GPCR antibodies can also be detected in various other disease patterns and in healthy individuals without the presence of fatigue symptoms.^35^ Interestingly, a recent placebo-controlled cross-over trial of rovunaptabin (BC007), a thrombin-binding aptamer, described slight improvements in PCS symptom severity, which was a secondary endpoint of the study.^36^ Rovunaptabin allegedly depletes GPCR autoantibodies. However, autoantibodies disappeared even after placebo treatment in that study, when symptom severity did not change.^36^

The participants of IAMPOCO had experienced PCS symptoms for a long time, averaging 21.6 months. It is possible that autoimmunity has caused irreversible damage at this point and that any treatment targeting autoantibodies can therefore no longer be effective. However, the majority of PCS patients experienced their primary infection during the first years of the pandemic, but are now looking for viable treatment options.^4^ Our study cannot therefore rule out a potential benefit of IA in early PCS.

Another possible reason why no clinical benefit was observed is the time frame of the study. Many autoantibody-mediated conditions for which immunoadsorption or therapeutic plasma exchange are established treatments, a clinical improvement is observed within days, often even after the first treatment.^19^^,^^37^

The analysis was done “per protocol”. Some subjects elected not to undergo a second treatment cycle and did not present for further follow-up visits, precluding any intention-to-treat analysis. Interestingly, 5 subjects dropped out after an initial round of immunoadsorption treatments, but none of these indicated an improvement in clinical condition as the reason for leaving the study. On the other hand, both of the 2 subjects that left the study after their initial sham treatment reported an improvement and stated that they did not want to risk receiving what they mistakenly thought would be sham treatment in the second cycle (when they would have been treated with true immunoadsorption).

It is possible that the immunoadsorption performed in our study was not effective enough in depleting autoantibody levels to achieve a clinical response. We used tryptophan adsorbers for our study, where circulating immunoglobulins bind to a tryptophan-coated surface. Other adsorbers employ peptides or other molecules to achieve immunoglobulin binding. The adsorbent defines the specificity and possibly efficacy of adsorption. However, to our knowledge, no study has directly compared tryptophan with other adsorbents; all adsorbents are successfully used in clinical practice; and the efficacy of eliminating antibodies and probability of success appear do depend more on baseline antibody concentrations and patient characteristics than on the specific adsorbent that is used. Importantly, no adsorbent achieves total depletion of autoantibodies, and no clinical threshold concentrations have been established that predict the occurrence (or resolution) of clinical signs and symptoms in post-Covid syndrome. In this context, it should be noted that autoantibody ‘signatures’ have been described in conditions other than PCS and, importantly, also in healthy individuals.^11^

The percentage of subjects with three prior vaccinations against SARS-CoV-2 was higher in the group that received immunoadsorption first and sham treatment last than it was in the group that started with sham treatment. However, this likely does not affect the results of the study, as every subject who completed two cycles underwent both immunoadsorption and sham treatment in this crossover study.

Although immunoadsorption is generally considered a safe procedure, adverse events may occur that we also saw in our study. Of note, immunoadsorption but not sham treatment was associated with a few jugular vein thromboses and clotting of the extracorporeal lines, hinting at a pro-coagulant effect of the absorption. When considering immunoadsorption as a last resort for subjects that are extremely affected by PCS, these risks need to be taken into account.

IAMPOCO is the only randomised controlled trial of the therapeutic effect of an extracorporeal therapy on PCS severity in a cross-over design. PCS is a very heterogeneous condition with a multitude of described symptoms, making it difficult to compare individual patients with PCS.^9^ This problem was addressed in the study design with the cross-over design, in which each patient represents his or her own control, which is one of the great strengths of this study.

However, the study also has limitations. Patients with PCS suffer from a wide range of symptoms, some of which cannot be adequately recorded and mapped using questionnaires, so that improvements that do not affect fatigue or basal cognitive functions may not be mapped, especially since the selection of questionnaires focused on fatigue symptoms. Furthermore, in this study only patients and evaluators were blinded, so that a bias on the part of the treatment team cannot be ruled out. The follow-up period was set at 6 weeks after the respective treatments, so that changes in symptoms that occurred later might not have been recorded. Although experience with the treatment of antibody-mediated autoimmune diseases shows that treatment effects tend to occur within 10–14 days, it cannot be ruled out that there were delayed effects, which would have been noticed at least in the group of participants who were treated first with IA after the second treatment cycle. Patients with a previous psychiatric diagnosis were excluded from this study. Although there is data indicating that a subset of patients with PCS have a significant degree of psychiatric comorbidity, this exclusion criterion could limit the transferability of the results.^38^ However, the symptoms examined in our study, most notably fatigue, can also be caused by psychological disorders, meaning that IA cannot change the cause of the symptoms beyond a placebo effect. This would further compromise the interpretability of the study in the context of an already highly heterogeneous disease. Therefore, the study was designed to exclude patients with known psychiatric comorbidity.

Post-Covid syndrome is a very heterogeneous condition, and we expect future work to reveal subtypes and provide further insights into the pathophysiology. However, our study participants were exactly those patients that are currently being treated with extracorporeal therapies on a case-by-case basis, often paying for it themselves, and our aim was to provide a rational foundation (or not) for this practice.

In summary, this patient-blinded, sham-controlled cross-over trial did not find a tangible clinical benefit of immunoadsorption in post-Covid syndrome. While it is conceivable that antibody removal by a different technique may provide such benefit, there is no evidence from controlled studies to support the current practice to offer immunoadsorption to patients suffering from PCS. Understanding the pathophysiology of PCS and finding effective treatments remain pressing tasks.

## Contributors

MS: conceptualisation, methodology, data curation, writing–original draft, editing, and revisions, visualisation; AK: investigation, writing–editing; MM: investigation, sample analysis, writing–editing; SCBL: investigation; PC: investigation, writing–editing; VT: investigation, writing–editing; FR: data curation, writing–editing; LSL: investigation, writing–editing; PSW: data curation, writing–editing; IS: formal analysis, writing–editing, visualisation; AW: investigation, writing–editing; DK: conceptualisation, methodology, data curation, visualisation, writing–initial draft, editing, and revisions, supervision; JWM: conceptualisation, methodology, writing–initial draft, editing, and revisions, funding acquisition, project administration, supervision. All authors had full access to all the data in the study accept responsibility to submit for publication.

## Data sharing statement

The data collected as part of the study can be requested in written form from the corresponding author by qualified researchers with a legitimate interest. A written contract can then be drawn up to regulate the transfer of individual participant data that underlie the results reported in this article, after de-identification (text, tables, figures, and appendices) in accordance with the guidelines of the relevant ethics committee and funding institutions.

## Declaration of interests

The authors declare no potential conflicting interests.

## Appendix A Supplementary data

IAMPOCO TLRH R3 supplement

Statistical Analysis

Studienprotokoll IAMPOCO
