# Supplementary material for: Immunoadsorption Versus Sham Treatment for Post-COVID Syndrome: A Randomised Sham-Controlled Crossover Trial
Source: Lancet Reg Health Eur. 2026 Jun 11;67:101744. doi: 10.1016/j.lanepe.2026.101744 (PMC13272180; doi:10.1016/j.lanepe.2026.101744)
Supplement: IAMPOCO TLRH R3 supplement [file mmc1.docx]

**Immunoadsorption vs. sham treatment for post-Covid syndrome:
A randomised clinical trial**

Marco Stortz, Andreas Kommer, Myriam Meineck, Simone Cosima Boedecker-Lips, Paul Claßen, Vanessa Tomalla, Felix Rausch, Livia Sophie Lang, Philipp S. Wild, Irene Schmidtmann, Arndt Weinmann, Daniel Kraus, Julia Weinmann-Menke

Supplementary Appendix

[Supplementary Figure S1: Primary Outcomes over time 2](#_Toc228185704)

[Supplementary Figure S2: Serum IgG levels before and after IA and sham treatment 5](#_Toc228185705)

[Supplementary Figure S3: GPCR Aab concentrations over time 6](#_Toc228185706)

[Supplementary Table S1: Mixed model for ordinal endpoint PCFS 11](#_Toc228185707)

[Supplementary Table S2: Mixed model for ordinal endpoint PCFS with interaction 12](#_Toc228185708)

[Supplementary Table S3: Odds ratios for mixed model for ordinal endpoint PCFS with interaction 13](#_Toc228185709)

[Supplementary Table S4: Mixed model for MoCA 14](#_Toc228185710)

[Supplementary Table S5: Mean treatment effects in mixed model for MoCA 15](#_Toc228185711)

[Supplementary Table S6: Mixed model for MFI-20 16](#_Toc228185712)

[Supplementary Table S7: Mean treatment effects in mixed model for MFI-20 17](#_Toc228185713)

[Supplementary Table S8: Mixed model for Bell score 18](#_Toc228185714)

[Supplementary Table S9: Mean treatment effects in mixed model for Bell score 19](#_Toc228185715)

[Supplementary Table S10: Mixed model for Chalder fatigue scale 20](#_Toc228185716)

[Supplementary Table S11: Mean treatment effects in mixed model for Chalder fatigue scale 21](#_Toc228185717)

[Supplementary Table S12: Mixed model for grip strength 22](#_Toc228185718)

[Supplementary Table S13: Mean treatment effects in mixed model for grip strength 23](#_Toc228185719)

[Supplementary Table S14: Adverse Events 24](#_Toc228185720)

[Supplementary Table S15: Relative levels of autoantibodies before and after IA and sham treatment, respectively 25](#_Toc228185721)

# Supplementary Figure S1: Primary Outcomes over time

Time-Points:

- T1 = “Start of 1st therapy cycle = week 0”
- T2 = “End of 1st therapy cycle = week 1”
- T3 = “14 d after 1st therapy cycle = week 3”
- T4 = “Start of 2nd therapy cycle = 6 weeks after 1st cycle = week 9”
- T5 = “End of 2nd therapy cycle = week 10”
- T6 = “14 d after 2nd therapy cycle = week 12”
- T7 = “6 weeks after 2nd cycle = week 16”

## A) Change in PCFS over time by treatment sequence

**
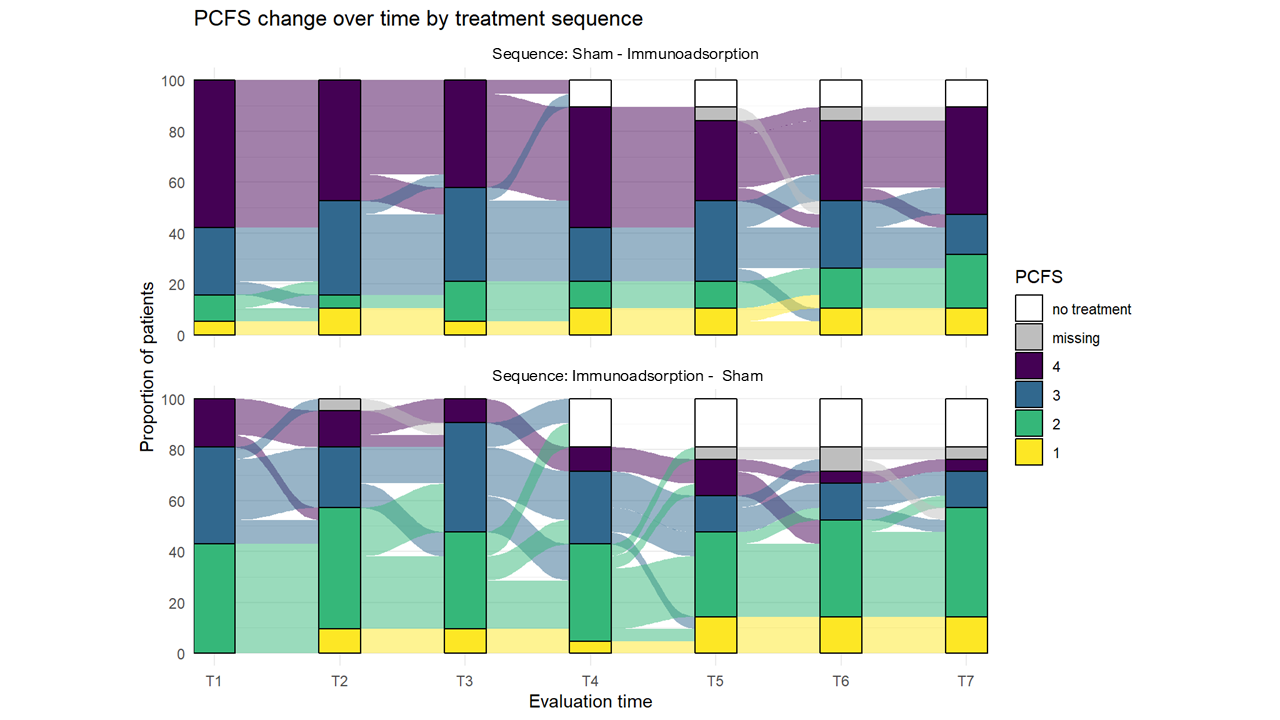
**

## B) Change in MoCA over time by treatment sequence

**
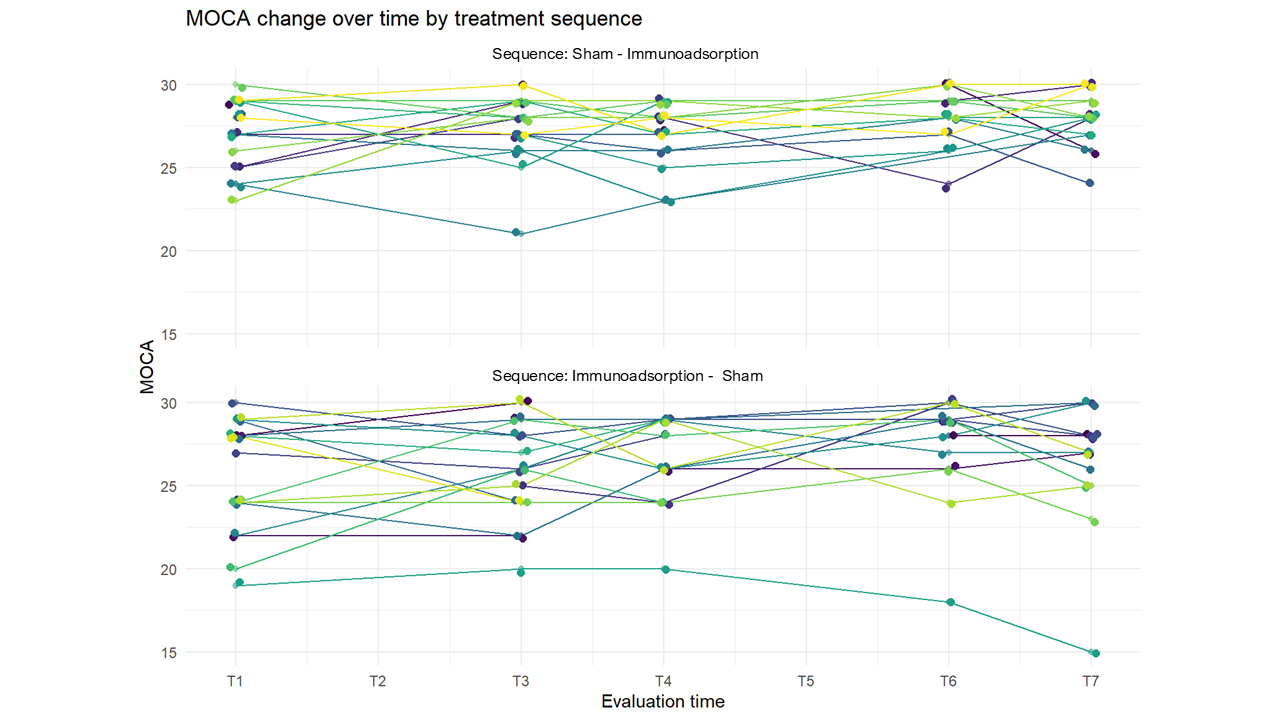
**

## C) Change in MFI-20 over time by treatment sequence


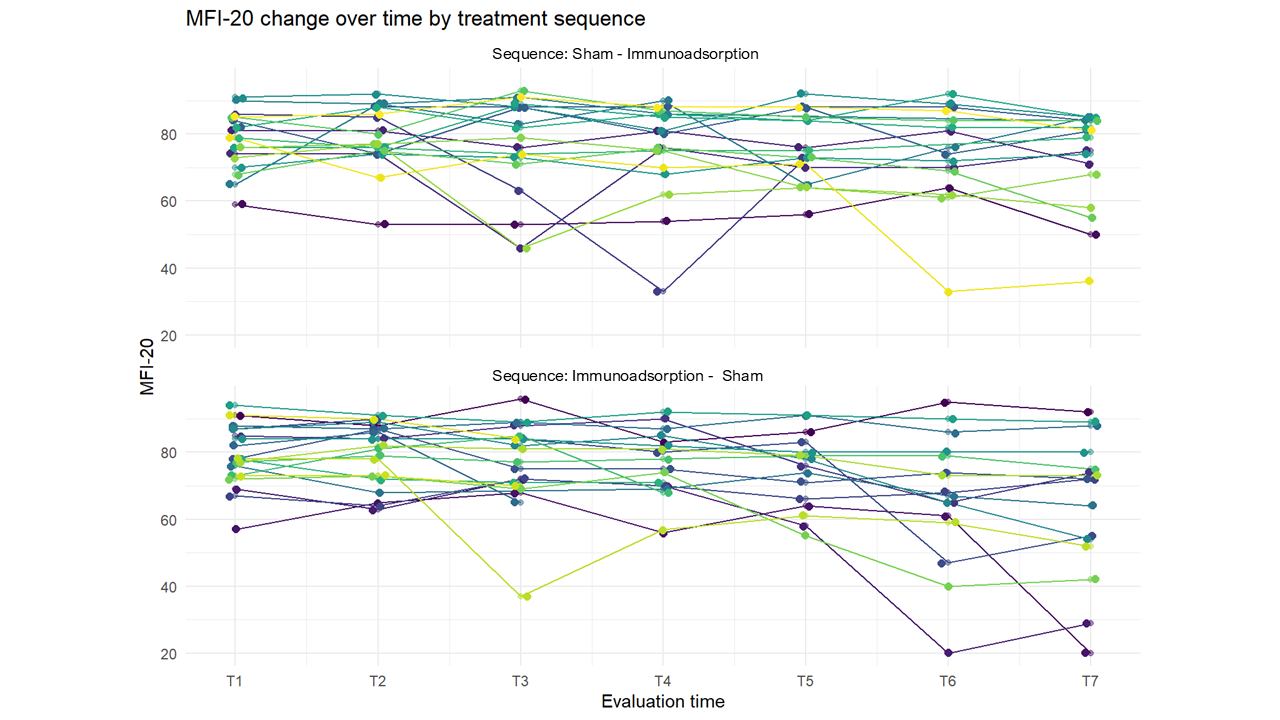


## D) Change in Bell score over time by treatment sequence


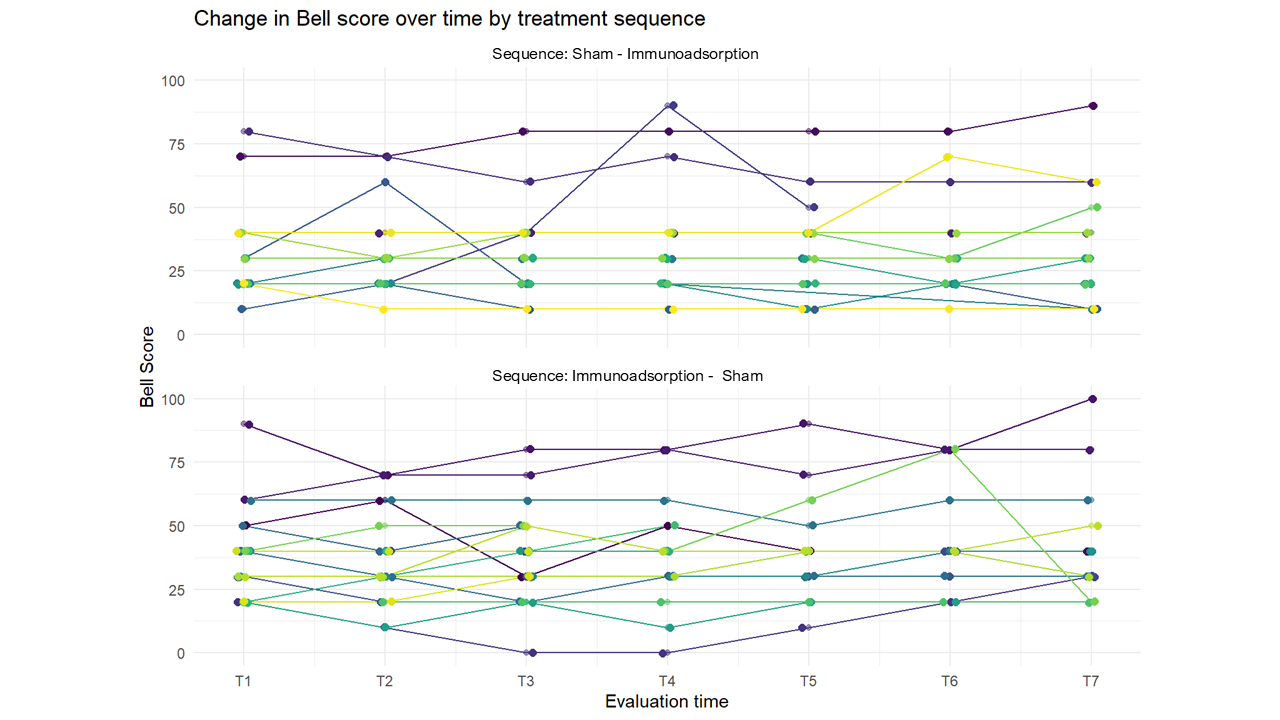


## E) Change in Chalder score over time by treatment sequence


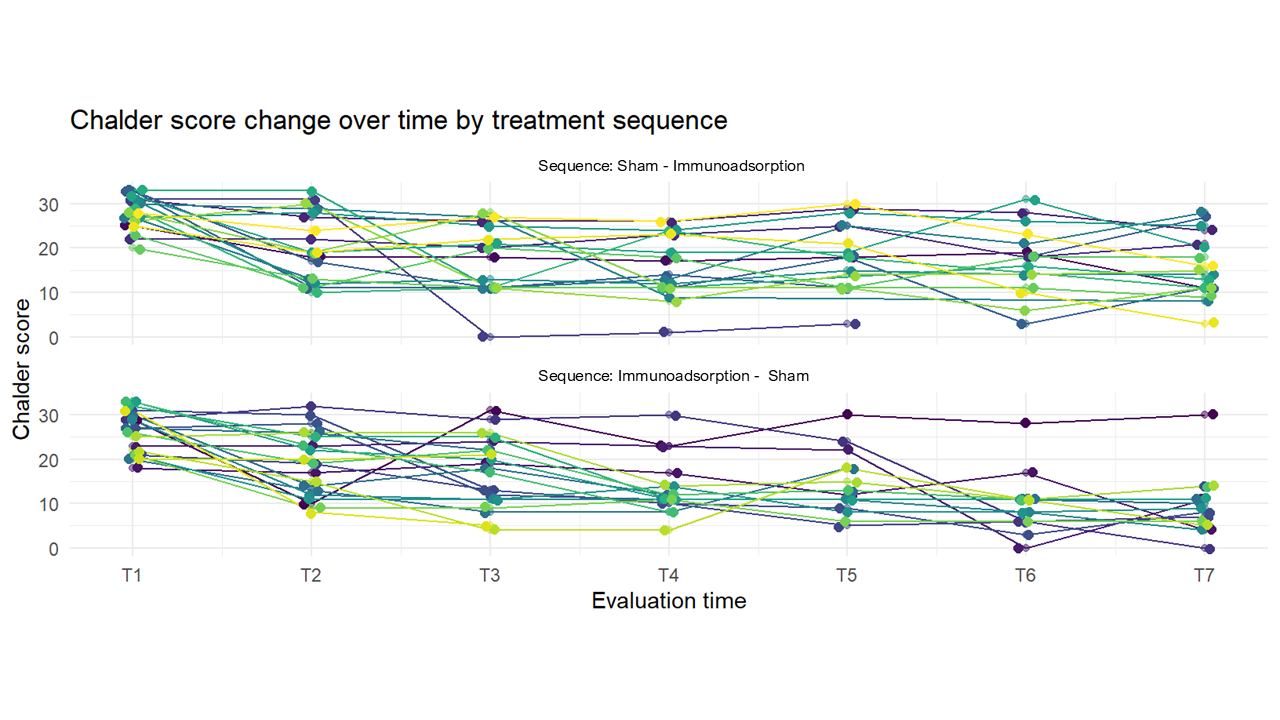


## F) Change in hand-grip strength over time by treatment sequence


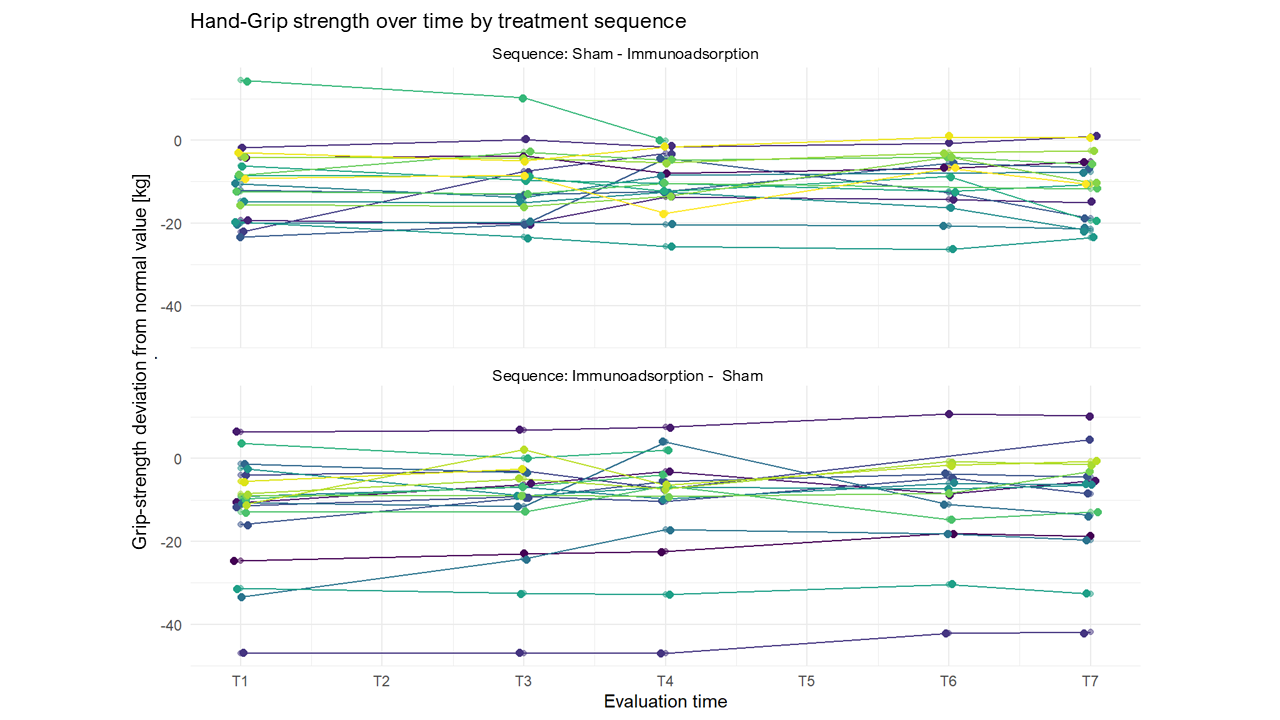


# Supplementary Figure S2: Serum IgG levels before and after IA and sham treatment


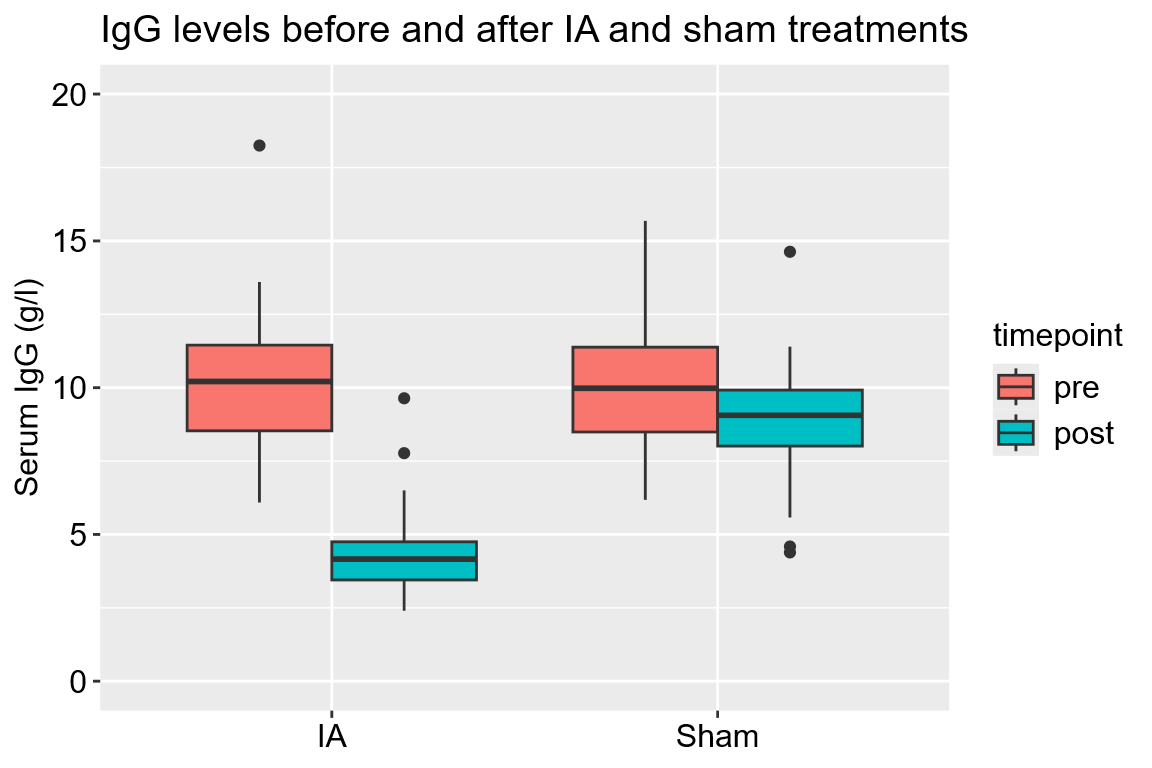


# Supplementary Figure S3: GPCR Aab concentrations over time

Time points:

- T1 = “Start of 1st therapy cycle = week 0”
- T2 = “End of 1st therapy cycle = week 1”
- T4 = “Start of 2nd therapy cycle = 6 weeks after 1st cycle = week 9”
- T5 = “End of 2nd therapy cycle = week 10”

## A) M1 Aabs over time by treatment sequence

**
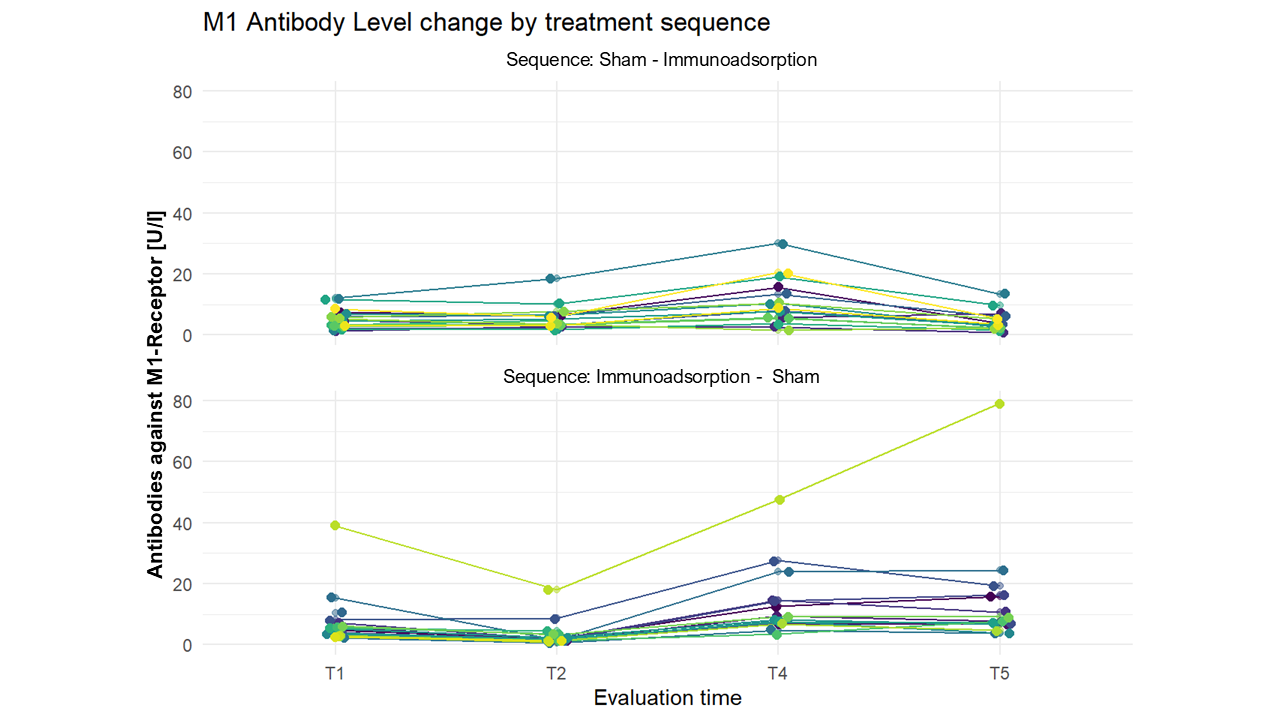
**

## B) M2 Aabs over time by treatment sequence


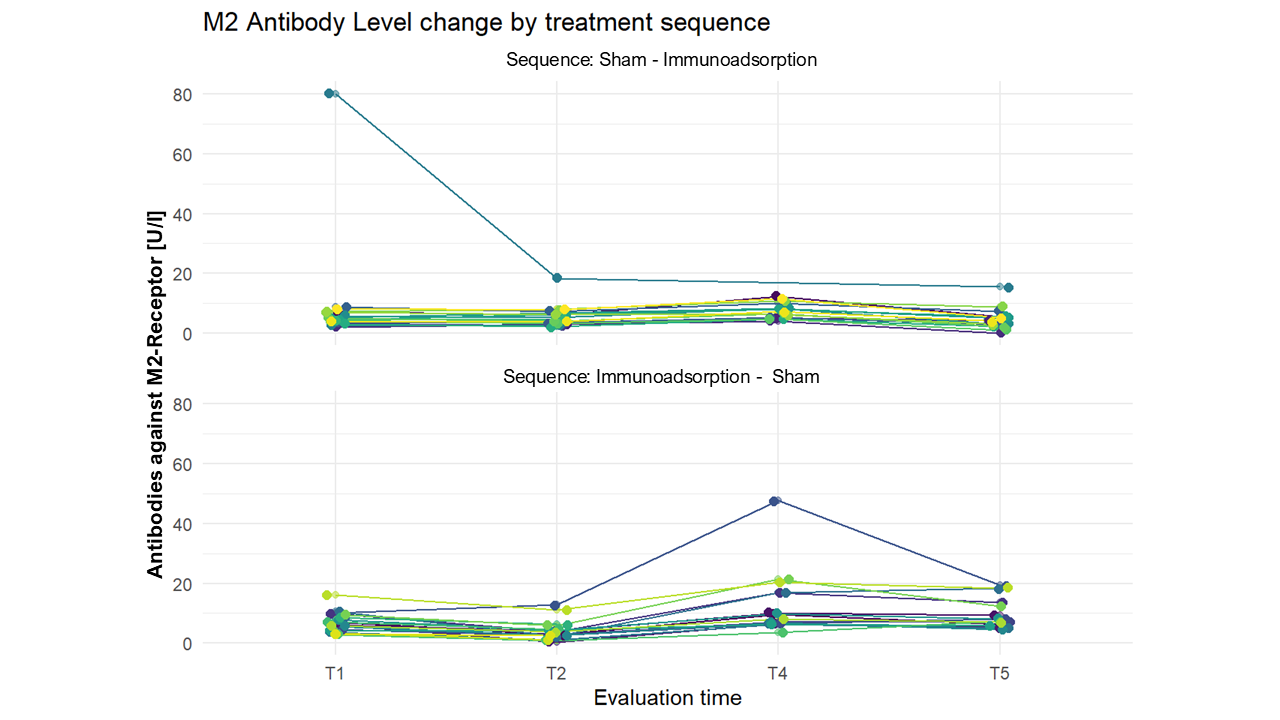


## C) M3 Aabs over time by treatment sequence


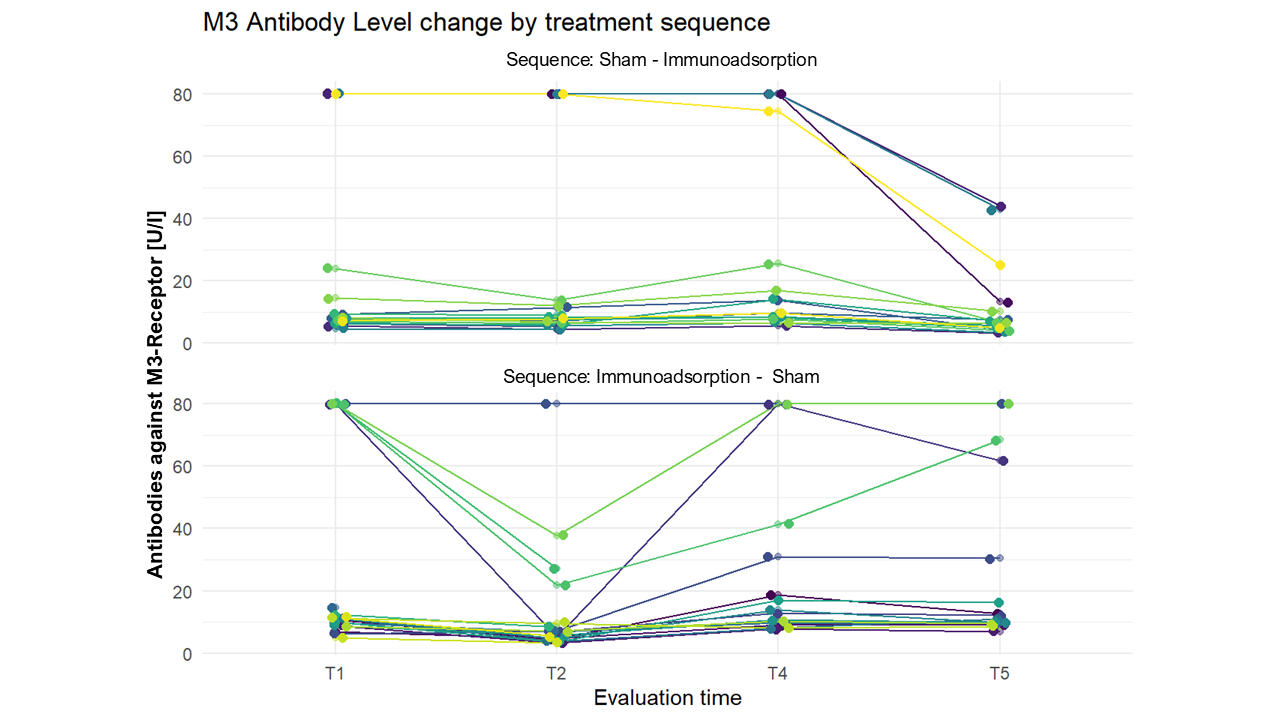


## D) M4 Aabs over time by treatment sequence


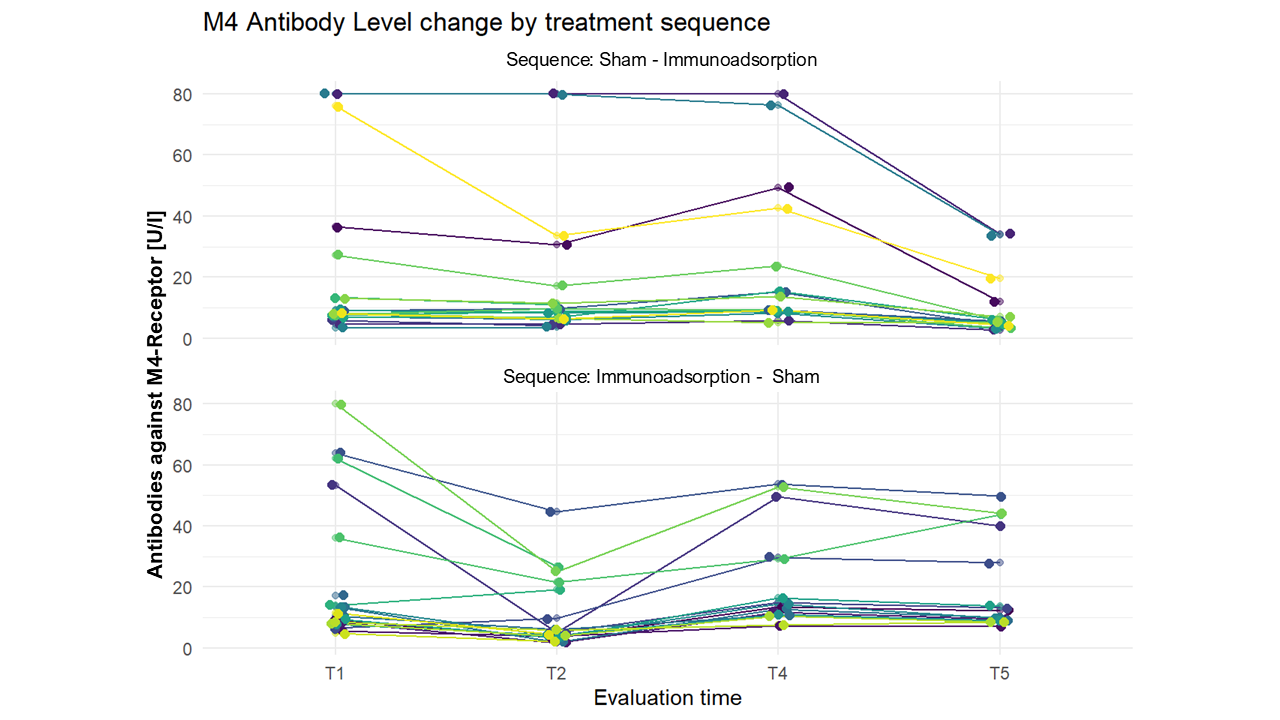


## E) M5 Aabs over time by treatment sequence


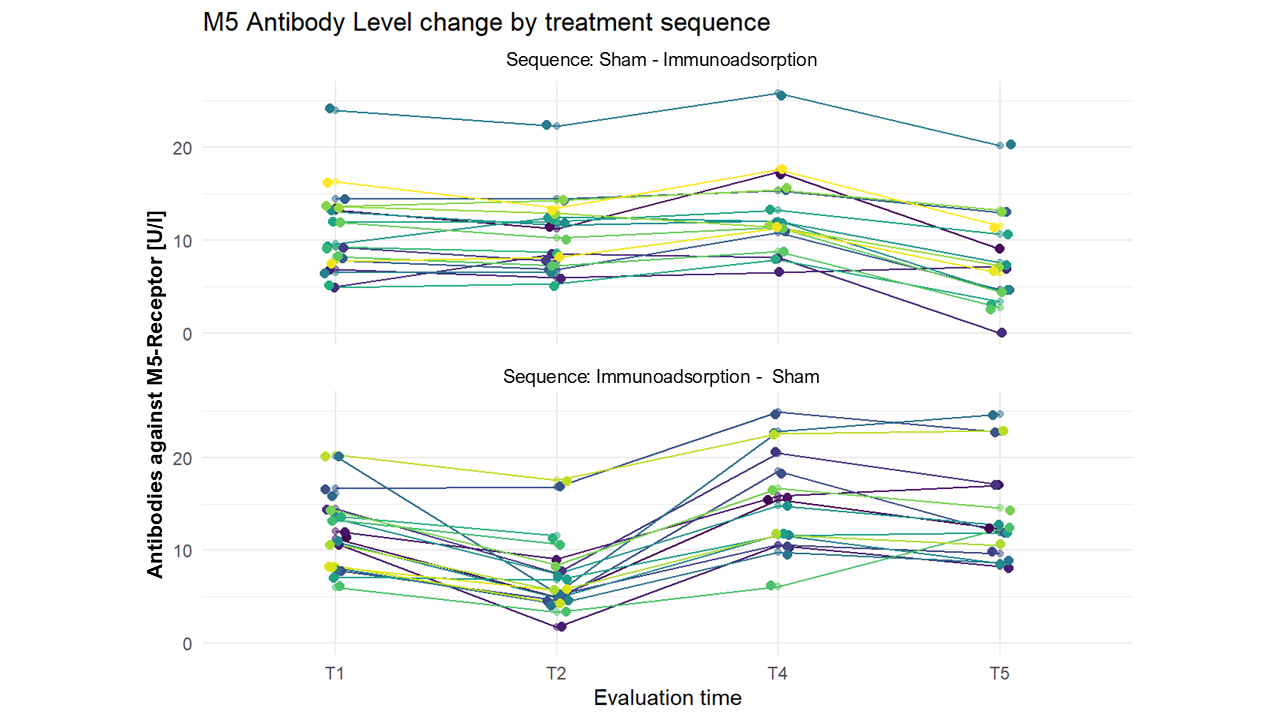


## F) Alpha-1 Aabs over time by treatment sequence


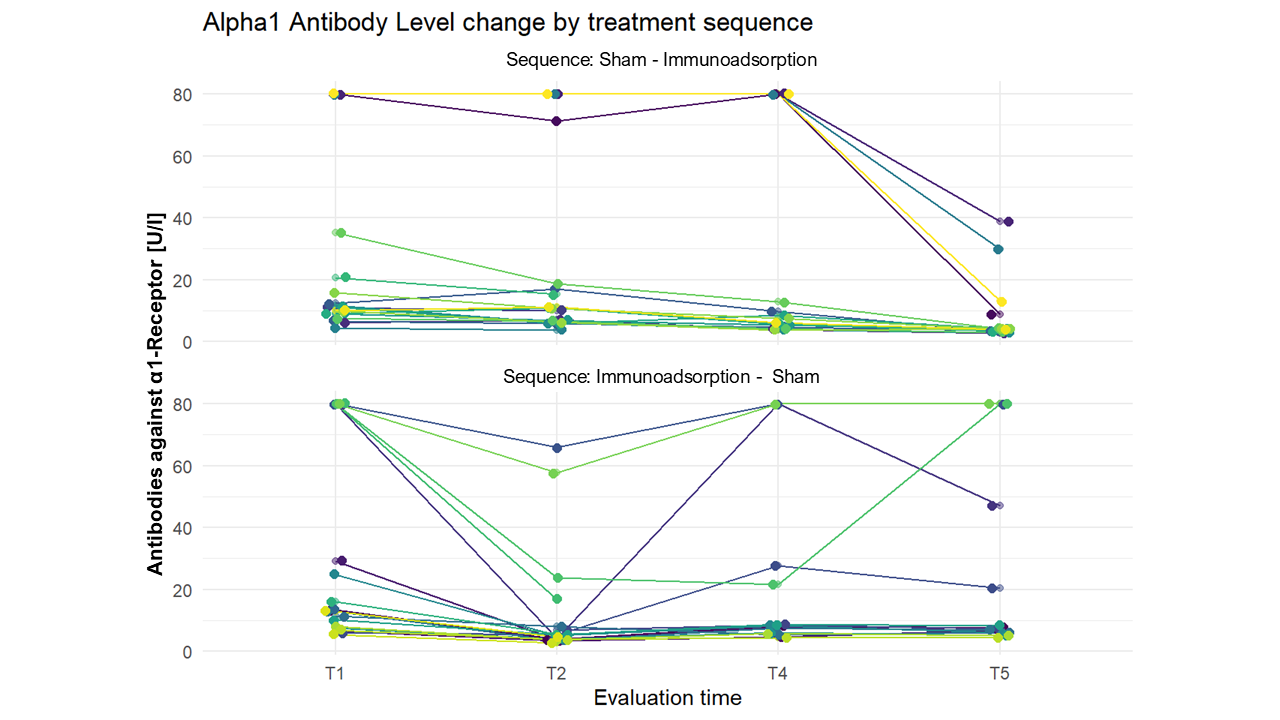


## G) Alpha-2 Aabs over time by treatment sequence


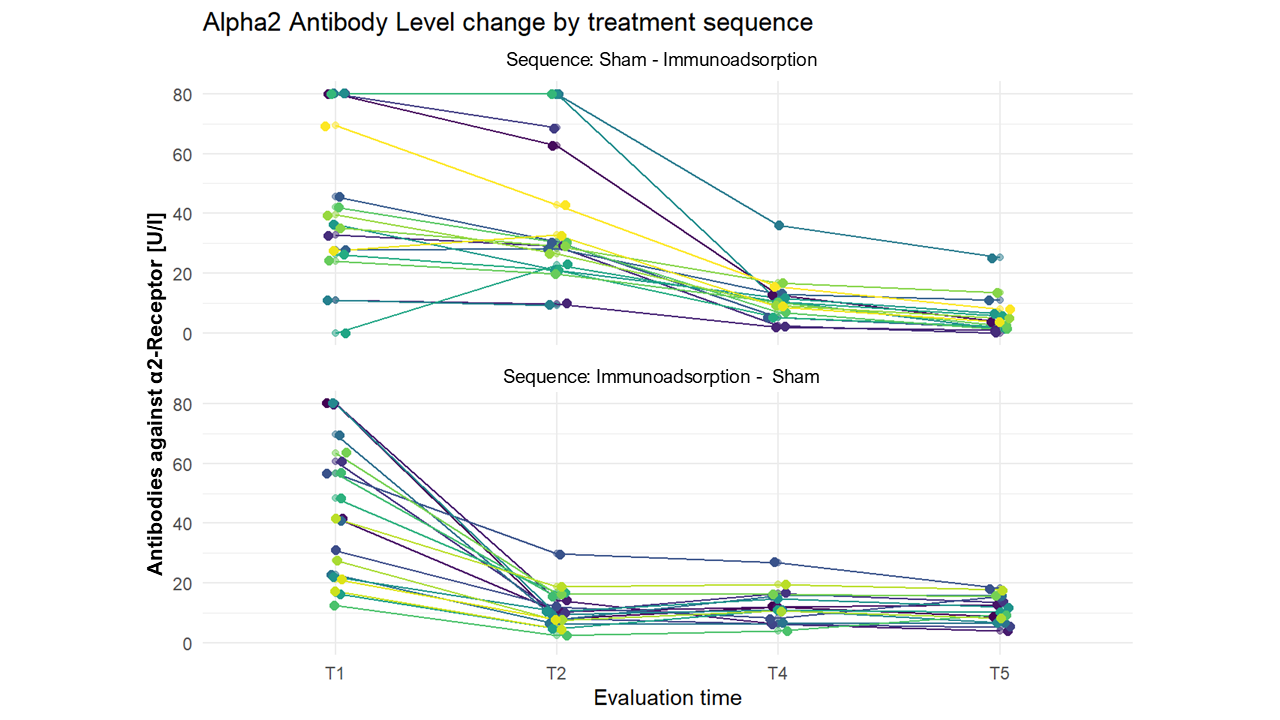


## H) Beta-1 Aabs over time by treatment sequence


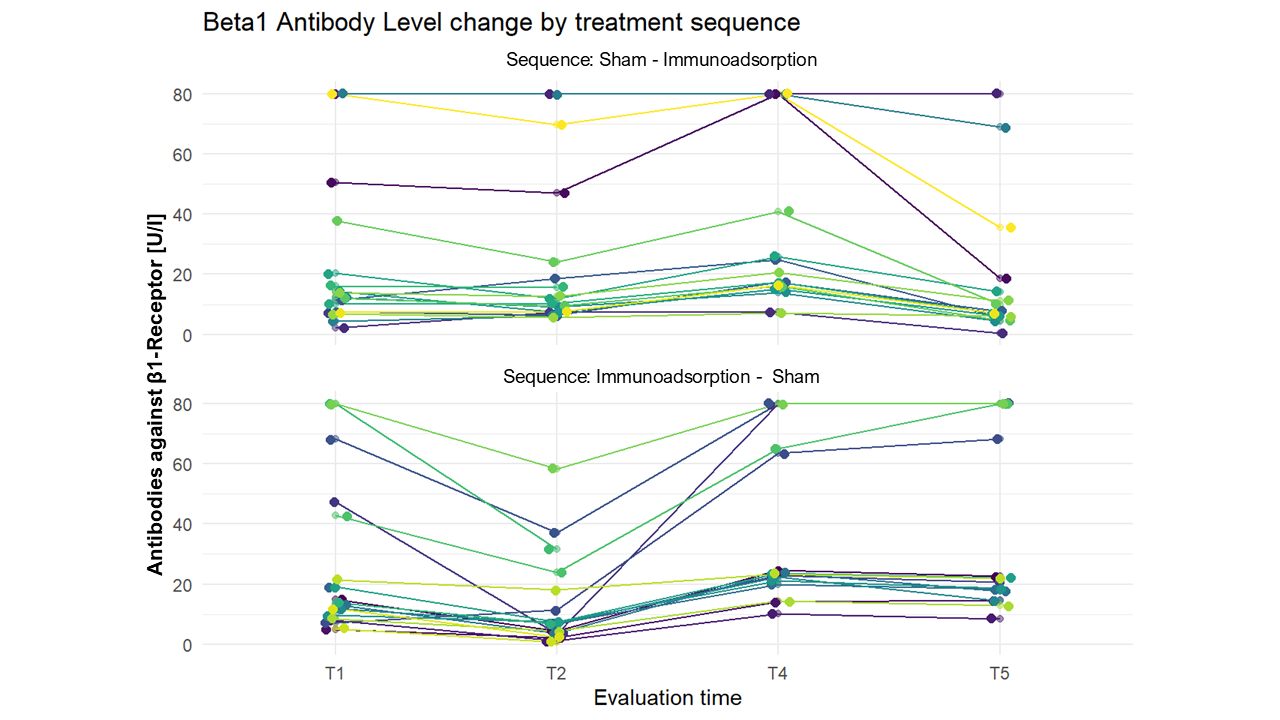


## I) Beta-2 Aabs over time by treatment sequence


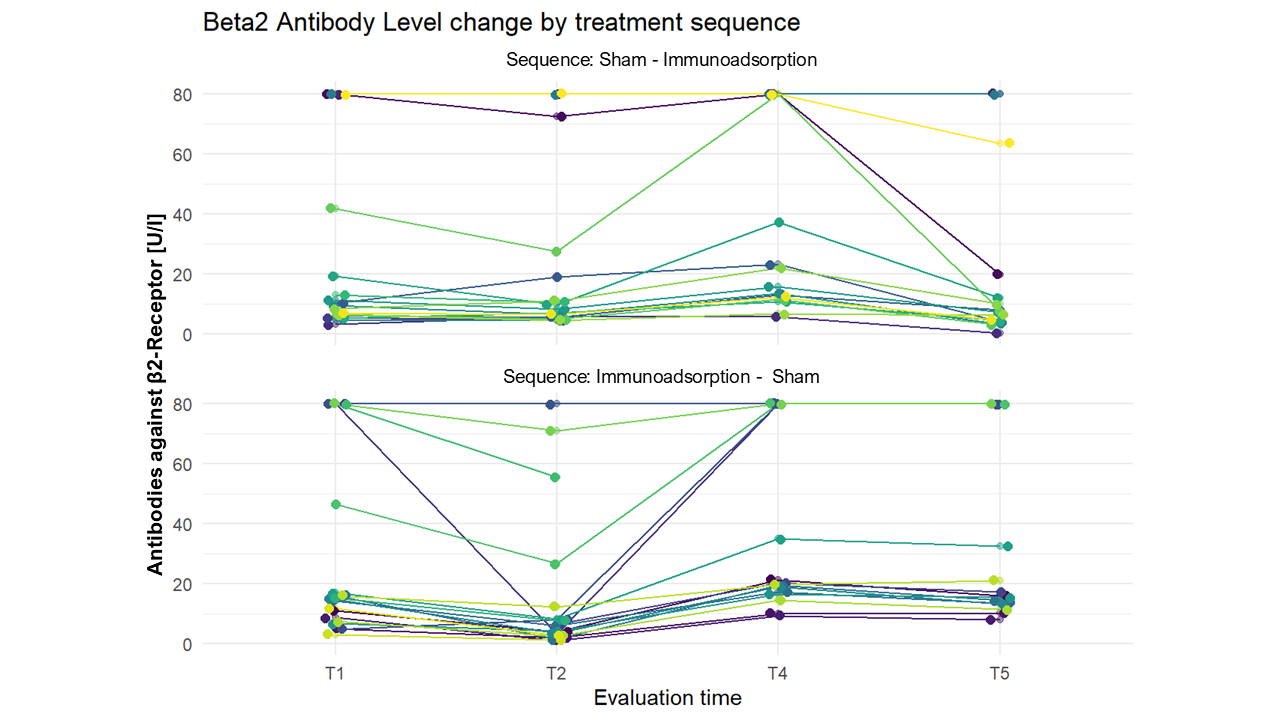


# Supplementary Table S1: Mixed model for ordinal endpoint PCFS

| Characteristic | OR | 95% CI | p-value | OR | 95% CI | p-value |
| --- | --- | --- | --- | --- | --- | --- |
| Treatment |  |  |  |  |  |  |
| Sham | — | — |  | — | — |  |
| IA | 1·17 | 0·41, 3·36 | 0·771 | 1·29 | 0·45, 3·67 | 0·636 |
| Treatment cycle |  |  |  |  |  |  |
| 1 | — | — |  | — | — |  |
| 2 | 1·16 | 0·40, 3·35 | 0·785 | 1·31 | 0·46, 3·76 | 0·611 |
| Course of index infection |  |  |  |  |  |  |
| mild |  |  |  | — | — |  |
| severe |  |  |  | 0·24 | 0·07, 0·88 | 0·031 |
| Age |  |  |  | 1·02 | 0·98, 1·07 | 0·337 |
| Duration of disease [months] |  |  |  | 1·00 | 0·94, 1·07 | 0·984 |
| Infection after which symptoms started |  |  |  | 0·39 | 0·02, 6·48 | 0·514 |
| Total number of infections |  |  |  | 2·33 | 0·64, 8·52 | 0·199 |
| Vaccination |  |  |  | 0·60 | 0·30, 1·21 | 0·153 |

Exploratory analyses to account for potential differences in age, duration of post-Covid syndrome, course of primary SARS-CoV-2 infection (index infection), number of infections, and vaccination status. These variables were included as covariates in a mixed model. In the original dataset, the course of the index infection was recorded as one of five levels: asymptomatic, at home with mild symptoms, at home with severe symptoms, hospitalised, hospitalised requiring additional oxygen. Due to very low numbers in some of these levels, the variable was dichotomised into “mild” (including asymptomatic or at home with mild symptoms) and “severe” (including all other levels) for the purpose of this exploratory analysis.

# Supplementary Table S2: Mixed model for ordinal endpoint PCFS with interaction

| Characteristic | Regression coefficient β | 95% CI | p-value |
| --- | --- | --- | --- |
| Treatment |  |  |  |
| Sham | — | — |  |
| IA | 1·9 | 0·15, 3·6 | 0·034 |
| Course of index infection |  |  |  |
| mild | — | — |  |
| severe | 0·06 | -1·7, 1·9 | 0·948 |
| Treatment cycle |  |  |  |
| 1 | — | — |  |
| 2 | 0·38 | -0·76, 1·5 | 0·517 |
| Age | 0·03 | -0·03, 0·08 | 0·331 |
| Duration of disease [months] | -0·01 | -0·08, 0·07 | 0·874 |
| Infection after which symptoms started | -1·3 | -4·6, 1·9 | 0·422 |
| Total number of infections | 1·1 | -0·40, 2·6 | 0·149 |
| Vaccination | -0·58 | -1·4, 0·22 | 0·155 |
| Treatment * Course of index infection |  |  |  |
| IA * severe | -3·4 | -6·0, -0·72 | 0·013 |

Exploratory analyses to account for potential differences in age, duration of post-Covid syndrome, course of primary SARS-CoV-2 infection (index infection), number of infections, and vaccination status. These variables were included as covariates in a mixed model. In the original dataset, the course of the index infection was recorded as one of five levels: asymptomatic, at home with mild symptoms, at home with severe symptoms, hospitalised, hospitalised requiring additional oxygen. Due to very low numbers in some of these levels, the variable was dichotomised into “mild” (including asymptomatic or at home with mild symptoms) and “severe” (including all other levels) for the purpose of this exploratory analysis.

# Supplementary Table S3: Odds ratios for mixed model for ordinal endpoint PCFS with interaction

| Course of index infection | comparison | OR | CI | p value |
| --- | --- | --- | --- | --- |
| mild | IA vs Sham | 6·63 | 1·16, 38 | 0·034 |
| severe | IA vs Sham | 0·23 | 0·04, 1·35 | 0·104 |

Exploratory analyses to account for potential differences in age, duration of post-Covid syndrome, course of primary SARS-CoV-2 infection (index infection), number of infections, and vaccination status. These variables were included as covariates in a mixed model. In the original dataset, the course of the index infection was recorded as one of five levels: asymptomatic, at home with mild symptoms, at home with severe symptoms, hospitalised, hospitalised requiring additional oxygen. Due to very low numbers in some of these levels, the variable was dichotomised into “mild” (including asymptomatic or at home with mild symptoms) and “severe” (including all other levels) for the purpose of this exploratory analysis.

# Supplementary Table S4: Mixed model for MoCA

| Covariate | Beta | 95% CI | p-value | Beta | 95% CI | p-value |
| --- | --- | --- | --- | --- | --- | --- |
| Treatment |  |  |  |  |  |  |
| Sham |  |  |  |  |  |  |
| IA | -0·01 | -1·2, 1·1 | 0·993 | -0·13 | -1·2, 0·97 | 0·819 |
| Treatment cycle |  |  |  |  |  |  |
| 1 |  |  |  |  |  |  |
| 2 | 0·56 | -0·59, 1·7 | 0·341 | 0·55 | -0·57, 1·7 | 0·343 |
| id.sd__(Intercept) | 0·00 |  |  | 0·00 |  |  |
| Residual.sd__Observation | 2·4 |  |  | 2·3 |  |  |
| Course of index infection |  |  |  |  |  |  |
| mild |  |  |  |  |  |  |
| severe |  |  |  | 0·79 | -0·38, 2·0 | 0·192 |
| Age |  |  |  | -0·02 | -0·06, 0·02 | 0·350 |
| Duration of disease [months] |  |  |  | -0·04 | -0·11, 0·03 | 0·252 |
| Infection after which symptoms started |  |  |  | 0·59 | -2·2, 3·4 | 0·677 |
| Total number of infections |  |  |  | -0·91 | -2·2, 0·37 | 0·169 |
| Vaccination |  |  |  | 0·57 | -0·08, 1·2 | 0·091 |

Exploratory analyses to account for potential differences in age, duration of post-Covid syndrome, course of primary SARS-CoV-2 infection (index infection), number of infections, and vaccination status. These variables were included as covariates in a mixed model. In the original dataset, the course of the index infection was recorded as one of five levels: asymptomatic, at home with mild symptoms, at home with severe symptoms, hospitalised, hospitalised requiring additional oxygen. Due to very low numbers in some of these levels, the variable was dichotomised into “mild” (including asymptomatic or at home with mild symptoms) and “severe” (including all other levels) for the purpose of this exploratory analysis.

# Supplementary Table S5: Mean treatment effects in mixed model for MoCA

| treatment | emmean | SE | df | lower.CL | upper.CL |
| --- | --- | --- | --- | --- | --- |
| Sham | 0·7111367 | 0·4099114 | 60·50257 | -0·1086682 | 1·530942 |
| IA | 0·5817869 | 0·3947424 | 60·48416 | -0·2076856 | 1·371259 |

# Supplementary Table S6: Mixed model for MFI-20

| Covariate | Beta | 95% CI | p-value | Beta | 95% CI | p-value |
| --- | --- | --- | --- | --- | --- | --- |
| Treatment |  |  |  |  |  |  |
| Sham |  |  |  |  |  |  |
| IA | 2·4 | -3·7, 8·5 | 0·437 | 2·5 | -3·8, 8·8 | 0·439 |
| Treatment cycle |  |  |  |  |  |  |
| 1 |  |  |  |  |  |  |
| 2 | -4·7 | -11, 1·4 | 0·136 | -4·9 | -11, 1·5 | 0·136 |
| id.sd__(Intercept) | 0·00 |  |  | 0·00 |  |  |
| Residual.sd__Observation | 13 |  |  | 13 |  |  |
| Course of index infection |  |  |  |  |  |  |
| mild |  |  |  |  |  |  |
| severe |  |  |  | -0·14 | -6·8, 6·5 | 0·968 |
| Age |  |  |  | 0·06 | -0·18, 0·31 | 0·611 |
| Duration of disease [months] |  |  |  | 0·13 | -0·23, 0·49 | 0·484 |
| Infection after which symptoms started |  |  |  | -3·6 | -19, 12 | 0·654 |
| Total number of infections |  |  |  | 2·3 | -4·8, 9·4 | 0·529 |
| Vaccination |  |  |  | 1·1 | -2·6, 4·8 | 0·555 |

Exploratory analyses to account for potential differences in age, duration of post-Covid syndrome, course of primary SARS-CoV-2 infection (index infection), number of infections, and vaccination status. These variables were included as covariates in a mixed model. In the original dataset, the course of the index infection was recorded as one of five levels: asymptomatic, at home with mild symptoms, at home with severe symptoms, hospitalised, hospitalised requiring additional oxygen. Due to very low numbers in some of these levels, the variable was dichotomised into “mild” (including asymptomatic or at home with mild symptoms) and “severe” (including all other levels) for the purpose of this exploratory analysis.

# Supplementary Table S7: Mean treatment effects in mixed model for MFI-20

| treatment | emmean | SE | df | lower.CL | upper.CL |
| --- | --- | --- | --- | --- | --- |
| Sham | -5·886888 | 2·306953 | 61·42857 | -10·499275 | -1·274500 |
| IA | -3·385816 | 2·280455 | 61·49591 | -7·945123 | 1·173492 |

# Supplementary Table S8: Mixed model for Bell score

| Covariate | Beta | 95% CI | p-value | Beta | 95% CI | p-value |
| --- | --- | --- | --- | --- | --- | --- |
| Treatment |  |  |  |  |  |  |
| Sham |  |  |  |  |  |  |
| IA | -2·6 | -6·9, 1·8 | 0·246 | -2·8 | -7·2, 1·6 | 0·217 |
| Treatment cycle |  |  |  |  |  |  |
| 1 |  |  |  |  |  |  |
| 2 | 3·0 | -1·4, 7·4 | 0·182 | 3·1 | -1·3, 7·5 | 0·177 |
| id.sd__(Intercept) | 4·1 |  |  | 4·4 |  |  |
| Residual.sd__Observation | 9·3 |  |  | 9·4 |  |  |
| Course of index infection |  |  |  |  |  |  |
| mild |  |  |  |  |  |  |
| severe |  |  |  | 0·77 | -4·6, 6·2 | 0·781 |
| Age |  |  |  | -0·17 | -0·37, 0·03 | 0·108 |
| Duration of disease [months] |  |  |  | -0·08 | -0·38, 0·21 | 0·589 |
| Infection after which symptoms started |  |  |  | 2·0 | -11, 15 | 0·758 |
| Total number of infections |  |  |  | -1·4 | -7·2, 4·4 | 0·633 |
| Vaccination |  |  |  | 1·5 | -1·6, 4·5 | 0·348 |

Exploratory analyses to account for potential differences in age, duration of post-Covid syndrome, course of primary SARS-CoV-2 infection (index infection), number of infections, and vaccination status. These variables were included as covariates in a mixed model. In the original dataset, the course of the index infection was recorded as one of five levels: asymptomatic, at home with mild symptoms, at home with severe symptoms, hospitalised, hospitalised requiring additional oxygen. Due to very low numbers in some of these levels, the variable was dichotomised into “mild” (including asymptomatic or at home with mild symptoms) and “severe” (including all other levels) for the purpose of this exploratory analysis.

# Supplementary Table S9: Mean treatment effects in mixed model for Bell score

| treatment | emmean | SE | df | lower.CL | upper.CL |
| --- | --- | --- | --- | --- | --- |
| Sham | 3·0373215 | 1·769928 | 60·09869 | -0·5029416 | 6·577585 |
| IA | 0·2411491 | 1·722954 | 59·80116 | -3·2055085 | 3·687807 |

# Supplementary Table S10: Mixed model for Chalder fatigue scale

| Covariate | Beta | 95% CI | p-value | Beta | 95% CI | p-value |
| --- | --- | --- | --- | --- | --- | --- |
| Treatment |  |  |  |  |  |  |
| Sham |  |  |  |  |  |  |
| IA | 0·09 | -4·5, 4·7 | 0·970 | 0·91 | -4·3, 6·1 | 0·734 |
| Treatment cycle |  |  |  |  |  |  |
| 1 |  |  |  |  |  |  |
| 2 | -7·0 | -12, -2·1 | 0·007 | -6·0 | -12, -0·46 | 0·038 |
| Treatment * Treatment cycle |  |  |  |  |  |  |
| IA * 2 | 6·8 | -1·1, 15 | 0·094 | 5·0 | -4·3, 14 | 0·296 |
| id.sd__(Intercept) | 4·4 |  |  | 4·7 |  |  |
| Residual.sd__Observation | 6·0 |  |  | 6·0 |  |  |
| Course of index infection |  |  |  |  |  |  |
| mild |  |  |  |  |  |  |
| severe |  |  |  | -1·4 | -5·7, 2·8 | 0·512 |
| Age |  |  |  | -0·06 | -0·23, 0·10 | 0·469 |
| Duration of disease [months] |  |  |  | 0·08 | -0·16, 0·31 | 0·511 |
| Infection after which symptoms started |  |  |  | -3·1 | -13, 7·0 | 0·549 |
| Total number of infections |  |  |  | 2·3 | -2·4, 7·0 | 0·338 |
| Vaccination |  |  |  | 0·58 | -1·8, 3·0 | 0·631 |

Exploratory analyses to account for potential differences in age, duration of post-Covid syndrome, course of primary SARS-CoV-2 infection (index infection), number of infections, and vaccination status. These variables were included as covariates in a mixed model. In the original dataset, the course of the index infection was recorded as one of five levels: asymptomatic, at home with mild symptoms, at home with severe symptoms, hospitalised, hospitalised requiring additional oxygen. Due to very low numbers in some of these levels, the variable was dichotomised into “mild” (including asymptomatic or at home with mild symptoms) and “severe” (including all other levels) for the purpose of this exploratory analysis.

# Supplementary Table S11: Mean treatment effects in mixed model for Chalder fatigue scale

| treatment | emmean | SE | df | lower.CL | upper.CL |
| --- | --- | --- | --- | --- | --- |
| Sham | 13·54214 | 1·258145 | 64·23114 | 11·02888 | 16·05539 |
| IA | 17·05192 | 1·232249 | 63·41931 | 14·58978 | 19·51405 |

# Supplementary Table S12: Mixed model for grip strength

| Covariate | Beta | 95% CI | p-value | Beta | 95% CI | p-value |
| --- | --- | --- | --- | --- | --- | --- |
| Treatment |  |  |  |  |  |  |
| Sham |  |  |  |  |  |  |
| IA | 1·3 | -0·83, 3·5 | 0·234 | 1·4 | -0·76, 3·5 | 0·209 |
| Treatment cycle |  |  |  |  |  |  |
| 1 |  |  |  |  |  |  |
| 2 | -0·08 | -2·2, 2·1 | 0·945 | -0·16 | -2·3, 2·0 | 0·889 |
| id.sd__(Intercept) | 1·6 |  |  | 2·2 |  |  |
| Residual.sd__Observation | 4·5 |  |  | 4·5 |  |  |
| Course of index infection |  |  |  |  |  |  |
| mild |  |  |  |  |  |  |
| severe |  |  |  | 0·31 | -2·4, 3·0 | 0·824 |
| Age |  |  |  | 0·02 | -0·08, 0·12 | 0·667 |
| Duration of disease [months] |  |  |  | 0·02 | -0·15, 0·18 | 0·855 |
| Infection after which symptoms started |  |  |  | 0·74 | -5·7, 7·2 | 0·822 |
| Total number of infections |  |  |  | 0·76 | -2·2, 3·7 | 0·616 |
| Vaccination |  |  |  | -0·01 | -1·5, 1·5 | 0·985 |

Exploratory analyses to account for potential differences in age, duration of post-Covid syndrome, course of primary SARS-CoV-2 infection (index infection), number of infections, and vaccination status. These variables were included as covariates in a mixed model. In the original dataset, the course of the index infection was recorded as one of five levels: asymptomatic, at home with mild symptoms, at home with severe symptoms, hospitalised, hospitalised requiring additional oxygen. Due to very low numbers in some of these levels, the variable was dichotomised into “mild” (including asymptomatic or at home with mild symptoms) and “severe” (including all other levels) for the purpose of this exploratory analysis.

# Supplementary Table S13: Mean treatment effects in mixed model for grip strength

| treatment | emmean | SE | df | lower.CL | upper.CL |
| --- | --- | --- | --- | --- | --- |
| Sham | 0·3653327 | 0·8678315 | 57·10858 | -1·3723983 | 2·103064 |
| IA | 1·7604742 | 0·8531204 | 56·98010 | 0·0521171 | 3·468831 |

# Supplementary Table S14: Adverse Events

|  | | **Immunoadsorption** | **Sham treatment** |
| --- | --- | --- | --- |
| mild | Clotting | 3 |  |
|  | Virus reactivation | 1 | 2 |
|  | Headache | 1 | 1 |
|  | Peripheral venous puncture problems | 1 | 1 |
|  | iron deficiency | 1 | 3 |
|  | Blood leak in the separator | 1 |  |
|  | Paresthesia | 1 |  |
|  | Mild infection after therapy cycle | 2 |  |
|  | Central venous puncture problems | 2 |  |
|  | Skin rash | 1 | 1 |
|  | Air bubbles in the hose system |  | 1 |
|  | Total | 14 | 9 |
| moderate | Drop in blood pressure | 2 |  |
|  | Hypocalcemia | 1 |  |
|  | Anemia | 1 |  |
|  | Syncope | 1 | 1 |
|  | Crash of fatigue | 2 |  |
|  | jugular vein thrombosis | 3 |  |
|  | Total | 10 | 1 |

# Supplementary Table S15: Relative levels of autoantibodies before and after IA and sham treatment, respectively

| Antibody |  | Ratio after/before | | Relative change [%] | |
| --- | --- | --- | --- | --- | --- |
|  | Baseline | Immunoadsorption | Sham | Immunoadsorption | Sham |
| m1 | 6·1 (6·3) | 0·48 (0·27) | 1·06 (0·36) | -52 (27) | 6 (36) |
| m2 | 8·0 (12·2) | 0·54 (0·26) | 0·92 (0·31) | -46 (26) | -8 (31) |
| m3 | 25·7 (30·3) | 0·54 (0·24) | 0·96 (0·19) | -46 (24) | -4 (19) |
| m4 | 22·0 (24·6) | 0·50 (0·29) | 0·90 (0·19) | -50 (29) | -10 (19) |
| m5 | 11·6 (4·3) | 0·60 (0·24) | 0·98 (0·25) | -40 (24) | -2 (25) |
| a1 | 27·8 (29·6) | 0·49 (0·25) | 0·99 (0·58) | -51 (25) | -1 (58) |
| a2 | 44·1 (24·5) | 0·35 (0·20) | 0·92 (0·35) | -65 (20) | -8 (35) |
| beta1 | 25·0 (25·5) | 0·47 (0·30) | 1·00 (0·47) | -53 (30) | 0 (47) |
| beta2 | 26·0 (29·1) | 0·49 (0·35) | 0·95 (0·28) | -51 (35) | -5 (28) |
| Values denote means of individual ratios (after/before) and percentages ([after-before]/before * 100), and values in parentheses denote standard deviation. | | | | | |
