# Supplementary material for: Immunoadsorption Versus Sham Treatment for Post-COVID Syndrome: A Randomised Sham-Controlled Crossover Trial
Source: Lancet Reg Health Eur. 2026 Jun 11;67:101744. doi: 10.1016/j.lanepe.2026.101744 (PMC13272180; doi:10.1016/j.lanepe.2026.101744)
Supplement: Statistical Analysis [file mmc2.docx]

Statistical analysis plan IAMPOCO

Table of contents

[1 Relevant points from the study protocol 42](#_Toc185748506)

[2 Estimands and analysis population 43](#_Toc185748508)

[2.1 Hypothetical strategy 43](#_Toc185748509)

[2.2 Principal stratum strategy 43](#_Toc185748510)

[3 Planned analyses 43](#_Toc185748511)

[3.1 Demographics 43](#_Toc185748512)

[3.2 Analysis points 44](#_Toc185748513)

[3.3 Analysis of primary target values 44](#_Toc185748514)

[3.3.1 Quantitative target values 44](#_Toc185748515)

[3.3.2 PCFS 45](#_Toc185748516)

[3.4 Analysis of secondary target figures 45](#_Toc185748517)

[3.4.1 Autoantibodies 45](#_Toc185748518)

[3.4.2 Subscales 46](#_Toc185748519)

[3.4.3 General well-being 46](#_Toc185748520)

[3.4.4 Adverse events 46](#_Toc185748521)

[3.5 Clinical data 46](#_Toc185748522)

[3.6 Laboratory parameters 46](#_Toc185748523)

[3.6.1 Characterization of patients 47](#_Toc185748524)

[3.6.2 Effects under therapy 47](#_Toc185748525)

[3.6.3 Vital parameters 48](#_Toc185748526)

[3.7 Association between anti-autologous antibody concentration and symptom severity 48](#_Toc185748527)

[3.7.1 Baseline 48](#_Toc185748528)

[3.7.2 Changes 48](#_Toc185748529)

[4 Missing values 48](#_Toc185748530)

[5 Software 48](#_Toc185748531)

[6 Literature 49](#_Toc185748532)

Immunoadsorption study in Mainz in adults with post-COVID syndrome to evaluate the therapeutic effect on symptom burden – Statistical analysis plan

Version 1.2 02/23/2025

The study is described in more detail in the study protocol version 3 dated February 6, 2023, and in the manuscript accompanying the study protocol.

# Relevant points from the study protocol

- Prospective, randomized, single-blind, placebo-controlled study
- N = 40 total
- Crossover design
  - Sequence group A: 5 verum – 5 sham treatments
  - Sequence group B: 5 sham treatments – 5 verum


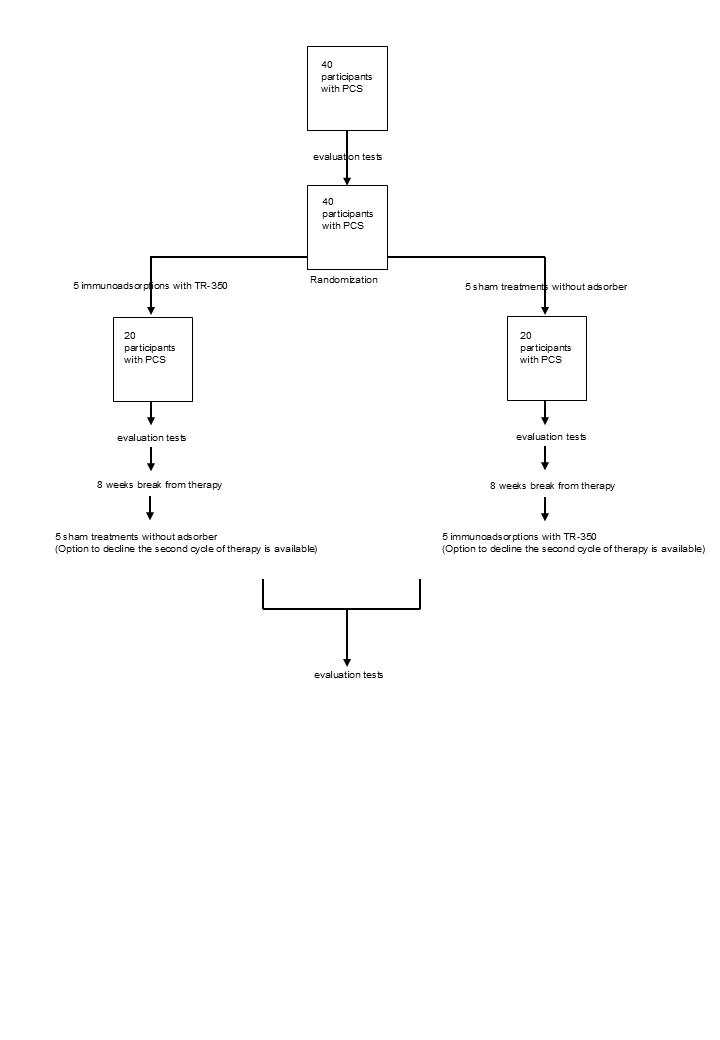


- Primary endpoint: Effectiveness of immune adsorption on symptom burden in patients with post-COVID syndrome measured using the values of:
  - Post-COVID-19 functional status scale (PCFS)
  - Chalder Fatigue Scale
  - Montreal Cognitive Assessment (MOCA)
  - MFI-20
  - Bell score
  - Strength measurement
- Secondary endpoints
  - Frequency of adverse events under active and sham treatment
  - Prevalence of anti-adrenergic and antimuscarinic autoantibodies in patients with post-COVID syndrome
  - Changes in the concentration of anti-adrenergic and anti-muscarinic autoantibodies under active and sham treatment

# Estimands and analysis population

The main intercurrent event that may occur in this study is discontinuation of the study after the first treatment cycle. Two strategies that take this into account are used in the analysis.

## Hypothetical strategy

The primary strategy will be a so-called "hypothetical strategy." Here, all randomized patients will be included with the treatment cycles in which they participated. This means that patients who only participated in the first treatment cycle and then discontinued their participation in the study remain in the analysis population. This corresponds to a hypothetical strategy and provides an estimator for the situation in which all patients participate in the study as planned – provided that the dropouts are "missing at random." "Missing at random" means that the fact that patients do not participate in the second treatment cycle depends only on previously observed values and not on the values they would have had in the second treatment cycle if they had participated. This assumption cannot be verified, but it seems plausible that this is the case.

## Principal stratum strategy

A principal stratum strategy is considered as a sensitivity analysis. Here, only patients who participated in both treatment cycles are included in the analysis. This provides the stratum of the population that tolerates both treatments regardless of the treatment sequence to which they were assigned. This is therefore a complete case analysis. In this case, the strategy corresponds to a classic per protocol analysis.

The two strategies differ only in whether or not patients who discontinued the study after the first treatment cycle are included in the analysis.

# Planned analyses

For both the "hypothetical strategy" and the "principal stratum strategy," the analyses described below are performed for the primary endpoints.

If more than 10% of patients did not participate in the second treatment cycle, a parallel group comparison after the first treatment cycle is also performed for the primary endpoints as a sensitivity analysis.

## Demographics

The following characteristics are used to characterize the patients and are described as follows. All data are determined for the total population and stratified by sequence group.

| **Characteristic** | **Measure** |
| --- | --- |
| Age | Mean, standard deviation, median, min, max, quartiles |
| Gender | Absolute and relative frequencies |
| Ethnicity | Absolute and relative frequencies |
| Smoking status | Absolute and relative frequencies, characteristics Smoker/ex-smoker/non-smoker |
| Highest level of education | Absolute and relative frequencies |
| Marital status | Absolute and relative frequencies, characteristics: single, married, divorced |
| Children | Absolute and relative frequencies for number, characteristics: 0/1/2/≥ 3 |
| Current occupation | Absolute and relative frequencies, characteristics: employed yes/no. |
| Occupation prior to SARS-COV-2 infection | Absolute and relative frequencies, characteristics employed yes/no. |

## Analysis points

The following terms are used to describe the time points for data collection in the analysis:

- T1= Start of therapy cycle 1 (week 0)
- T2 = end of therapy cycle 1 (week 1)
- T3 = 14 days after therapy cycle 1 (week 3)
- T4= Start of therapy cycle 2 (week 9)
- T5= End of therapy cycle 2 (week 10)
- T6 = 14 days after therapy cycle 2 (week 12)
- T7 = 6-8 weeks after therapy cycle 2 (week 16-18)

## Analysis of primary endpoints

The five primary endpoints listed below are considered equivalent. Adjustment for multiple testing is performed using the Bonferroni-Holm procedure. The test decision is based on the p-value for the main effect of the treatment (see belowmade ).

To compare effectiveness, the differences T3 – T1 and T6 – T4 are determined for the following characteristics.

- Post-COVID-19 functional status scale (PCFS)
- Montreal cognitive assessment (MOCA)
- MFI-20 (MFI-20 overall)
- Bell score
- Strength measurement

For the Chalder Fatigue Scale (Chalder overall), the values at T3 and T6 are considered, as this scale already describes a change from the baseline.

### Quantitative target values

With the exception of PCFS, the primary endpoints can be considered quantitative. Accordingly, the mean, standard deviation, median, min, max, and quartiles are determined, stratified by treatment (verum/placebo) and period (cycle 1/cycle 2). The tabular presentation is as follows

| Treatment | Period | Sequence group | Difference |
| --- | --- | --- | --- |
| Verum | Cycle 1 | A | T3 – T1 |
| Verum | Cycle 2 | B | T6 – T4 |
| Placebo | Cycle 1 | B | T3 – T1 |
| Placebo | Cycle 2 | A | T6 – T4 |

For the Chalder Fatigue Scale, the values for T3 and T6 are used instead of the differences.

A mixed linear model with treatment and period as fixed effects and patient as a random effect is used to evaluate the significance of the influence of treatment and period on the change under treatment. Marginal estimators and 95% confidence intervals for the effects of treatment and period are reported, and p-values for the main effects of treatment and period and for interactions are determined.

It is also examined whether a carry-over effect must be assumed. If the characteristics are clearly not normally distributed, a Mann-Whitney test for crossover is used.

### PCFS

For the ordinal characteristic PCFS, the absolute and relative frequencies of the individual values and their changes (by how many categories) are determined under treatment. In addition, analogous to the mixed linear model in3.3.1 , a generalized mixed linear model is used, which can be regarded as a generalization of an ordinal regression model. Treatment and period are also considered fixed effects and patient is considered a random effect.

## Analysis of secondary endpoints

### Autoantibodies

Analogous to the primary endpoints, the differences T2 – T1 and T5 – T4 are determined for the following antibodies to assess the effect of immunoadsorption.

- Concentration of anti-α1 adrenoreceptor antibodies
- Concentration of anti-α2-adrenoreceptor antibodies
- Concentration of anti-β1-adrenoreceptor antibodies
- Concentration of anti-β2-adrenoreceptor antibodies
- Concentration of anti-β3-adrenoreceptor antibodies
- Concentration of anti-M1 acetylcholine receptor antibodies
- Concentration of anti-M2 acetylcholine receptor antibodies
- Concentration of anti-M3 acetylcholine receptor antibodies
- Concentration of anti-M4 acetylcholine receptor antibodies

The absolute and relative frequency of patients in whom the antibody is detectable is indicated.

Furthermore, the mean, standard deviation, median, min, max, and quartiles are determined, stratified according to treatment (verum/placebo) and period (cycle 1/cycle 2). The tabular representation is as follows

| Treatment | Period | Sequence group | Difference |
| --- | --- | --- | --- |
| Verum | Cycle 1 | A | T2 – T1 |
| Verum | Cycle 2 | B | T5 – T4 |
| Placebo | Cycle 1 | B | T2 – T1 |
| Placebo | Cycle 2 | A | T5 – T4 |

- A mixed linear model with treatment and period as fixed effects and patient as a random effect is used to evaluate the significance of the influence of treatment and period on the change under treatment. Marginal estimators and 95% confidence intervals for the effects of treatment and period are reported, and p-values for the main effects of treatment and period and for interactions are determined.
- It is also examined whether a carry-over effect must be assumed. If the characteristics are clearly not normally distributed, a Mann-Whitney test for crossover is used.

### Subscales

The subscales of MFI-20 (MFI-20 general, MFI-20 physical, MFI-20 activity, MFI-20 motivation, MFI-20 psychic) and the Chalder Fatigue Scale (Chalder physical, Chalder mental) are evaluated in the same way as the overall scale.

### General well-being

For general well-being, the values at time points T3 and T6 are used for short-term effects and the values at time points T4 and T7 are used for long-term effects. The absolute and relative frequencies of the individual characteristics are determined. Comparisons are made using logistic regression for ordinal characteristics.

### Adverse events

Adverse events are recorded for up to six weeks after the end of each cycle. They are listed stratified by period and treatment. The highest severity grade is reported in each case.

Furthermore, the absolute and relative frequencies of at least one adverse event are reported stratified by period and treatment, thus determining the prevalence.

The frequency of discontinuation of therapy due to adverse events is also reported stratified by period and treatment.

## Clinical data

The following characteristics are used to characterize the patients and are described as follows. All data are determined for the total population and stratified by sequence group.

| **Characteristic** | **Calculation/measurement** |
| --- | --- |
| Pre-existing conditions | Absolute and relative frequencies |
| Time of SARS-COV-2 infection | Calculation of the time from diagnosis of SARS-CoV-2 infection to randomization |
| Vaccination status (number of vaccinations) in relation to SARS-CoV-2 | Absolute and relative frequencies |
| Symptoms during SARS-CoV-2 infection | Absolute and relative frequencies |
| Hospitalization status during SARS-CoV-2 infection | Absolute and relative frequencies, severity:  No, normal ward, intensive care unit |
| Need for oxygen administration during SARS-CoV-2 infection | Absolute and relative frequencies, characteristics:  Yes/No |

## Laboratory parameters

Laboratory parameters were collected at several time points (see3.2 ):

| **Parameter** | **Time of measurement** |
| --- | --- |
| Complete blood count   - Erythrocytes - Leukocytes - Thrombocytes - Neutrophil granulocytes - Eosinophil granulocytes - Basophilic granulocytes - Monocytes - Lymphocytes | T1, T3, T4, T6 |
| Sodium concentration | T1, T4 |
| Potassium concentration | T1, T4 |
| Calcium concentration | T1, T4 |
| Phosphate concentration | T1, T4 |
| Transferrin saturation | T1 |
| Ferritin | T1, T3, T4, T6 |
| C-reactive protein | T1, T3, T4, T6 |
| Antinuclear antibodies | T1, T3, T4, T6 |
| Thyroid-stimulating hormone | T1, T3, T4, T6 |
| 25-hydroxy vitamin D concentration | T |
| Vitamin B12 concentration | T |
| Immunoglobulin G concentration | T1, T3, T4, T6 |
| Activated partial thromboplastin time (aPTT) | T1, T4 |
| International normalized ratio (INR) | T1, T4 |
| fibrinogen concentration | T1, T3, T4, T6 |
| Albumin in spontaneous urine | T1, T4 |
| Creatinine in spontaneous urine | T1, T4 |
| Total protein in spontaneous urine | T1, T4 |
| Urine sediment including acanthocyte concentration | T1, T4 |
| Albumin/creatinine ratio in spontaneous urine | T1, T4 |
| Total protein/creatinine ratio in spontaneous urine | T1, T4 |
| C3 complement | T1, T3, T4, T6 |
| C4 complement | T1, T3, T4, T6 |
| Ch50 | T1, T3, T4, T6 |

### Characterization of patients

The values prior to therapy cycle 1 (T1) are used to characterize the patients. Mean, standard deviation, median, min, max, and quartiles are determined for the entire collective and stratified according to sequence group.

### Effects during therapy

The effects under therapy are described for the following laboratory parameters: CRP, fibrinogen concentration, C3 complement, C4 complement, Ch50.

To describe effects under therapy, the differences T3 – T1 and T6 – T4 are determined. Mean, standard deviation, median, min, max, and quartiles are determined, stratified by treatment (verum/placebo) and period (cycle 1/cycle 2). The tabular presentation is analogous to the presentation for the primary endpoints.

| Treatment | Period | Sequence group | Difference |
| --- | --- | --- | --- |
| Verum | Cycle 1 | A | T3 – T1 |
| Verum | Cycle 2 | B | T6 – T4 |
| Placebo | Cycle 1 | B | T3 – T1 |
| Placebo | Cycle 2 | A | T6 – T4 |

A mixed linear model with treatment and period as fixed effects and patient as a random effect is used to evaluate the significance of the influence of treatment and period on the change under treatment. Marginal estimators and 95% confidence intervals for the effects of treatment and period are reported, and p-values for the main effects of treatment and period and for interactions are determined.

If the distribution of the laboratory parameters deviates significantly from the normal distribution, a logarithmic transformation is performed.

### Vital parameters

To characterize the patients, the mean, standard deviation, median, min, max, and quartiles are determined for the following quantitative vital parameters for the entire collective and stratified according to sequence group.

- Body
- Weight
- BMI, calculated from height and weight (BMI = weight in kg / (height in m)^2^ )
- Systolic blood pressure
- Diastolic blood pressure
- Heart rate
- Body temperature
- Respiratory rate
- ECG
- MRC dyspnea scale

## Association between anti-autocrine concentration and symptom severity

### Baseline

To investigate whether there are associations between anti-autoantibody concentration and symptom burden, the correlations (Pearson correlation coefficient and Spearman correlation coefficient) between characteristics of the Post-COVID-19 Functional Status Scale (PCFS), Chalder Fatigue Scale, Montreal Cognitive Assessment (MOCA), MFI-20, and Bell score and autoantibody concentrations were determined.

Furthermore, regression models will be adjusted with the characteristics Post-COVID-19 Functional Status Scale (PCFS), Chalder Fatigue Scale, Montreal Cognitive Assessment (MOCA), MFI-20, and Bell Score as target variables and autoantibody concentrations as influencing variables. In a first step, a linear regression model will be adjusted to include the autoantibody concentrations that correlate significantly with the target variable at the 5% level as influencing factors. Further exploratory analyses may follow.

### Changes

Similarly, changes in symptom severity will be correlated with changes in autoantibody concentrations. To this end, the differences described in3.3 and3.4.1 will be determined. Correlations (Pearson correlation coefficient and Spearman correlation coefficient) will be determined and regression models will be adjusted. This analysis is also exploratory and not specified in detail a priori.

# Missing values

Multiple imputation is used to replace missing values in the primary and secondary endpoints. If patients withdraw from the study after the first treatment cycle, no values are replaced for the second treatment cycle.

# Software

The analyses are performed using SAS 9.4 or higher and/or R 4.4.1 or higher.

# Literature

Senn S. Cross-over Trials in Clinical Research. Second edition. Wiley 2002

Wellek S, Blettner M. On the proper use of the crossover design in clinical trials: part 18 of a series on evaluation of scientific publications. Dtsch Arztebl Int. 2012 Apr;109(15):276-81. doi: 10.3238/arztebl.2012.0276

Matthews JNS, Bazakou S, Henderson R, Sharples LD. Contrasting principal stratum and hypothetical strategy estimands in multi-period crossover trials with incomplete data. Biometrics. 2023 Sep;79(3):1896-1907. doi: 10.1111/biom.13777
