# Supplementary material for: Immunoadsorption Versus Sham Treatment for Post-COVID Syndrome: A Randomised Sham-Controlled Crossover Trial
Source: Lancet Reg Health Eur. 2026 Jun 11;67:101744. doi: 10.1016/j.lanepe.2026.101744 (PMC13272180; doi:10.1016/j.lanepe.2026.101744)
Supplement: Studienprotokoll IAMPOCO [file mmc3.docx]

# **Study protocol**

# **Immunoadsorption study in Mainz in adults with post-COVID syndrome to evaluate the therapeutic effect on symptom burden**

## **General**

## **Project title:** Investigation of the effect of Immunoadsorption therapy on symptom burden in adults with post-COVID syndrome

## **Version number:** 3.1

## **Version date:** February 27, 2022

## **Summary of the project**

| Study title | Mainz Immunoadsorption Study in Adults with Post-COVID (IAMPOCO) |
| --- | --- |
| Applic | Prof. Julia Weinmann-Menke, MD |
| Study location | Main |
| Fund | Third-party funding (Diamed Medizintechnik, Cologne, Germany) |
| Study design/type | Prospective randomized single-blind placebo-controlled study |
| Number of subjects | 40 subjects |
| Study population | Adults recruited from participants in the Gutenberg Post-COVID Study who had at least one positive SARS-CoV-2 PCR test at least 6 months prior to study inclusion, as well as new or persistent symptoms of SARS-CoV-2 infection 3 months after a positive PCR test that have persisted for at least 2 months. |
| Planned start of study | March 8, 2023 |
| Study duration per subject | 4.5 |
| Planned study duration | Recruitment period: 6 months  Duration of the clinical study: 18 months |
| Primary endpoint | Effectiveness of immunoadsorption on symptom burden in patients with post-COVID syndrome as measured by the values of:   - Post-COVID-19 Functional Status Scale (PCFS) - Chalder Fatigue Scale - Montreal Cognitive Assessment (MOCA) - MFI-20 - Bell Score - Strength measurement   And their comparison before and after verum therapy and before and after sham therapy (before therapy cycle 1/after therapy cycle 1 and before therapy cycle 2/after therapy cycle 2) |
| Secondary endpoints | 1. Frequency of adverse events under active and sham treatment  2. Prevalence of anti-adrenergic and anti-muscarinic autoantibodies in patients with post-COVID syndrome:   - Proportion of subjects with evidence of anti-α1-adrenoreceptor AK - Proportion of subjects with evidence of anti-α2-adrenergic receptor antibodies - Proportion of subjects with evidence of anti-β1-adrenoreceptor AK - Proportion of subjects with evidence of anti-β2-adrenoreceptor AK - Proportion of subjects with evidence of anti-β3-adrenoreceptor AK - Proportion of subjects with evidence of anti-M1 acetylcholine receptor AK - Proportion of subjects with evidence of anti-M2 acetylcholine receptor AK - Proportion of subjects with evidence of anti-M3 acetylcholine receptor AK - Proportion of subjects with evidence of anti-M4 acetylcholine receptor AK  1. Concentration of:  - Anti-α1 adrenoreceptor AK - Anti-α2-adrenoreceptor AK - Anti-β1 adrenoreceptor AK - Anti-β1 adrenoreceptor AK - Anti-β1-adrenoreceptor AK - Anti-M1 acetylcholine receptor AK - Anti-M2 acetylcholine receptor AK - Anti-M3 acetylcholine receptor AK - Anti-M4 acetylcholine receptor AK   Before and after treatment with active substance and placebo (before therapy cycle 1/after therapy cycle 1 and before therapy cycle 2/after therapy cycle 2) |
| Safety parameters | Not applicable |

## **Responsibilities**

## 3.1 Study director

**Prof. Julia Weinmann-Menke, MD**

I. Medical Clinic and Polyclinic

Focus on Rheumatology and Nephrology, University Medical Center Mainz

Langenbeckstraße 1

55131 Mainz

## [Julia.Weinmann-Menke@unimedizin-mainz.de](mailto:Julia.Weinmann-Menke@unimedizin-mainz.de)

## 3.2 Participating scientists

## **PD Dr. Arndt Weinmann**

I. Department of Medicine, University Medical Center of Johannes Gutenberg University Mainz

Langenbeckstr. 1

55131 Mainz

## **PD Dr. med. Daniel Kraus**

- I. Department of Medicine, University Medical Center of Johannes Gutenberg University Mainz

## Langenbeckstraße 1

## 55131 Mainz

- **Dr. Marco Stortz**
- I. Department of Medicine, University Medical Center of Johannes Gutenberg University Mainz

Langenbeckstraße 1

55131 Mainz

## 3.3 Participating institutions

- Nephrology Department of the First Medical Clinic and Polyclinic, University Medical Center Mainz
- Central Laboratory, University Medical Center Mainz
- Center for Cardiology, University Medical Center Mainz
- Clinic for Psychosomatic Medicine and Psychotherapy, University Medical Center Mainz

## 3.4 Funding

- Diamed GmbH, Cologne, Germany
- Treatment, inpatient stay, and experimental analyses are financed by the clinic's own funds.
- For detailed analyses such as RNAseq and proteome analyses, it is planned to obtain additional third-party funding where possible.

## 3.5 Registration in a publicly accessible study registry

Planned at clinicaltrials.gov

## **Scientific background**

Long or post-COVID refers to a syndrome of symptoms that develop three months after a suspected or confirmed infection with SARS-COV-2, have developed within the three months, or are still present from the acute infection and persist for at least two months.^1^ The type of symptoms does not play a role in the definition of post-COVID syndrome to date.^1, 2^ The prevalence of post-COVID syndrome is estimated at 43% of all SARS-COV-2 infected individuals, with hospitalized patients suffering from persistent symptoms more frequently (54%) than non-hospitalized patients (32%).^3^ Women are more likely to suffer from post-COVID syndromes, with a prevalence of 49%, than men, who are affected in 32% of cases of persistent or newly occurring symptoms after COVID-19, according to .^3^ The most common symptoms are fatigue (23% of those affected), memory problems (14%), dyspnea (13%), sleep disorders (11%), and joint pain (10%).^3^ Headaches, myalgia, anxiety, and depression are also frequently reported.^3^

In terms of the type, variety, and duration of symptoms, post-COVID resembles a clinical picture observed after various viral infections, such as the Epstein-Barr virus, the herpes simplex virus, or the influenza virus, namely myalgic encephalomyelitis and chronic fatigue syndrome (ME/CFS).^4, 5^ Here, too, patients mainly suffer from fatigue, concentration and memory problems, and non-restorative sleep.^4, 5^ Some authors see post-COVID as a form of ME/CFS triggered by SARS-COV-2 infection or the immune response to the infection.^5^

The possible pathomechanisms suspected to be behind these syndromes are diverse. However, the pathogenesis and underlying causes are not yet fully understood.^6^ Similarly, the causes of post-COVID syndrome are still unclear.^4^ Both clinical pictures are thought to involve autoimmune mechanisms triggered by the body's response to infection and sustained by the similarity of the body's own proteins to pathogen components (molecular mimicry).^7In^the context of this autoimmunity, antibodies against the body's own structures may also be formed, such as antinuclear antibodies, which target components of the cell nucleus.^7^ In patients suffering from ME/CFS, antibodies against α- and β-adrenergic receptors and against muscarinic acetylcholine receptors, among others, have been detected.^8^ No correlations between antibody concentrations and the severity of the disease or the clinical picture of ME/CFS could be demonstrated, nor are these antibodies specific or sensitive for ME/CFS.^8, 9^

With the detection of autoantibodies in patients with ME/CFS, forms of therapy that specifically target the formation of autoantibodies have also been investigated. For example, two randomized controlled trials involving 30 and 151 ME/CFS patients investigated B-cell-depleting therapy with the anti-CD-19 antibody rituximab versus placebo. The results showed no significant improvement in fatigue symptoms compared to placebo, although a reduction in B-cell concentration was achieved.^10, 11^ In the primary endpoints (improvement in fatigue symptoms after 3 months and after 24 months), there was no significant difference between placebo and rituximab therapy in either study.^10, 11^

Immunoadsorption is another therapeutic option for reducing the concentration of autoantibodies. In this procedure, the patient's blood is first separated into cellular components and plasma, and then the plasma is passed through adsorbents.^12^ The adsorbents have specific surfaces for binding different classes of immunoglobulins, so that the plasma is then largely purified of immunoglobulins and thus also of autoantibodies and returned to the patient together with the cellular components.^12^ This reduces the concentration of immunoglobulins and thus also the concentration of autoantibodies. ^12, 13^ Immunoadsorption is one of the established treatment options for many antibody-mediated autoimmune diseases.^14^

In patients with long COVID or post-COVID syndrome, there are no studies to date on possible effects on symptoms that are at least partially suspected to have an autoimmune origin. Only a small group of patients with the similar ME/CFS have been treated with immunoadsorption in a study, but without a control group or randomization and without objectively verifiable success.^15^

Due to the increasing number of people suffering from long COVID symptoms who are turning to doctors who cannot offer them any treatment or who offer treatments such as immunoadsorption without evidence, there appears to be an urgent need to investigate the effects of immunoadsorption on the course of long COVID syndrome.

1. Soriano JB, Murthy S, Marshall JC, et al. A clinical case definition of post-COVID-19 condition by a Delphi consensus. *Lancet Infect Dis* 2022; 22: e102-e107. 2021/12/25. DOI: 10.1016/s1473-3099(21)00703-9.

2. Koczulla AR, Ankermann T, Behrends U, et al. [S1 Guideline Post-COVID/Long-COVID]. *Pneumologie* 2021; 75: 869-900. 2021/09/03. DOI: 10.1055/a-1551-9734.

3. Chen C, Haupert SR, Zimmermann L, et al. Global Prevalence of Post COVID-19 Condition or Long COVID: A Meta-Analysis and Systematic Review. *J Infect Dis* 2022 2022/04/17. DOI: 10.1093/infdis/jiac136.

4. Poenaru S, Abdallah SJ, Corrales-Medina V, et al. COVID-19 and post-infectious myalgic encephalomyelitis/chronic fatigue syndrome: a narrative review. *Ther Adv Infect Dis* 2021; 8: 20499361211009385. 2021/05/08. DOI: 10.1177/20499361211009385.

5. Bateman L, Bested AC, Bonilla HF, et al. Myalgic Encephalomyelitis/Chronic Fatigue Syndrome: Essentials of Diagnosis and Management. *Mayo Clin Proc* 2021; 96: 2861-2878. 2021/08/30. DOI: 10.1016/j.mayocp.2021.07.004.

6. Rasa S, Nora-Krukle Z, Henning N, et al. Chronic viral infections in myalgic encephalomyelitis/chronic fatigue syndrome (ME/CFS). J *Transl Med* 2018; 16: 268. 2018/10/05. DOI: 10.1186/s12967-018-1644-y.

7. Woodruff MC, Ramonell RP, Saini AS, et al. Relaxed peripheral tolerance drives broad de novo autoreactivity in severe COVID-19. *medRxiv* 2021 2020/10/28. DOI: 10.1101/2020.10.21.20216192.

8. Bynke A, Julin P, Gottfries C-G, et al. Autoantibodies to beta-adrenergic and muscarinic cholinergic receptors in Myalgic Encephalomyelitis (ME) patients – A validation study in plasma and cerebrospinal fluid from two Swedish cohorts. *Brain, Behavior, &amp; Immunity - Health* 2020; 7: 100107. DOI: https://doi.org/10.1016/j.bbih.2020.100107.

9. Galloway A, Li H, Vanderlinde-Wood M, et al. Activating autoantibodies to the β1/2-adrenergic and M2 muscarinic receptors associate with atrial tachyarrhythmias in patients with hyperthyroidism. *Endocrine* 2015; 49: 457-463. 2014/12/17. DOI: 10.1007/s12020-014-0495-4.

10. Fluge Ø, Rekeland IG, Lien K, et al. B-Lymphocyte Depletion in Patients With Myalgic Encephalomyelitis/Chronic Fatigue Syndrome: A Randomized, Double-Blind, Placebo-Controlled Trial. *Ann Intern Med* 2019; 170: 585-593. 2019/04/02. DOI: 10.7326/m18-1451.

11. Fluge Ø, Bruland O, Risa K, et al. Benefit from B-lymphocyte depletion using the anti-CD20 antibody rituximab in chronic fatigue syndrome. A double-blind and placebo-controlled study. *PLoS One* 2011; 6: e26358. 2011/11/01. DOI: 10.1371/journal.pone.0026358.

12. Sułowicz W and Stompór T. LDL-apheresis and immunoadsorption: novel methods in the treatment of renal diseases refractory to conventional therapy. *Nephrol Dial Transplant* 2003; 18 Suppl 5: v59-62. 2003/06/21. DOI: 10.1093/ndt/gfg1050.

13. Boedecker SC, Luessi F, Engel S, et al. Immunoadsorption and plasma exchange—Efficient treatment options for neurological autoimmune diseases. *J Clin Apher* 2022; 37: 70-81. 2021/12/15. DOI: 10.1002/jca.21953.

14. Padmanabhan A, Connelly-Smith L, Aqui N, et al. Guidelines on the Use of Therapeutic Apheresis in Clinical Practice - Evidence-Based Approach from the Writing Committee of the American Society for Apheresis: The Eighth Special Issue. J *Clin Apher* 2019; 34: 171-354. 2019/06/11. DOI: 10.1002/jca.21705.

15. Scheibenbogen C, Loebel M, Freitag H, et al. Immunoadsorption to remove ß2 adrenergic receptor antibodies in Chronic Fatigue Syndrome CFS/ME. *PLoS One* 2018; 13: e0193672. 2018/03/16. DOI: 10.1371/journal.pone.0193672.

16. Klok FA, Boon G, Barco S, et al. The Post-COVID-19 Functional Status scale: a tool to measure functional status over time after COVID-19. *Eur Respir J* 2020; 56 2020/05/14. DOI: 10.1183/13993003.01494-2020.

17. Nasreddine ZS, Phillips NA, Bédirian V, et al. The Montreal Cognitive Assessment, MoCA: a brief screening tool for mild cognitive impairment. *J Am Geriatr Soc* 2005; 53: 695-699. 2005/04/09. DOI: 10.1111/j.1532-5415.2005.53221.x.

18. Bestall JC, Paul EA, Garrod R, et al. Usefulness of the Medical Research Council (MRC) dyspnea scale as a measure of disability in patients with chronic obstructive pulmonary disease. *Thorax* 1999; 54: 581-586. 1999/06/22. DOI: 10.1136/thx.54.7.581.

## **5. Project objectives**

- To examine the effects of immunoadsorption therapy on symptom burden in patients suffering from post-COVID symptoms
- Objective assessment of post-COVID symptoms using standardized tests and questionnaires
- To determine the prevalence of autoantibodies against alpha- and beta-adrenergic adrenoreceptors and muscarinic acetylcholine receptors, as well as possible correlations with certain post-COVID symptoms or their severity
- Verification of the effects of immunoadsorption therapy on the concentrations of autoantibodies against alpha- and beta-adrenergic adrenoreceptors and muscarinic acetylcholine receptors

## **6. Target parameters**

6.1 Primary endpoints:

Effectiveness of immunoadsorption on symptom burden in patients with post-COVID syndrome measured by the values of:

- Post-COVID-19 functional status scale (PCFS)
- Chalder Fatigue Scale
- Montreal Cognitive Assessment (MOCA)
- MFI-20
- Bell Score
- Strength measurement

and comparison before and after active treatment and before and after sham treatment (before treatment cycle 1/after treatment cycle 1 and before treatment cycle 2/after treatment cycle 2).

6.2 Secondary endpoints:

- Frequency of adverse events under active and sham treatment
- Prevalence of anti-adrenergic and anti-muscarinic autoantibodies in patients with post-COVID syndrome:
  - Proportion of subjects with evidence of anti-α1-adrenoreceptor Abs
  - Proportion of subjects with evidence of anti-α2-adrenergic receptor Abs
  - Proportion of subjects with evidence of anti-β1-adrenoreceptor Abs
  - Proportion of subjects with evidence of anti-β2-adrenergic receptor Abs
  - Proportion of subjects with evidence of anti-β3-adrenoreceptor Abs
  - Proportion of subjects with evidence of anti-M1 acetylcholine receptor Abs
  - Proportion of subjects with evidence of anti-M2 acetylcholine receptor Abs
  - Proportion of subjects with evidence of anti-M3 acetylcholine receptor Abs
  - Proportion of subjects with evidence of anti-M4 acetylcholine receptor Abs
- Concentration of:
  - Anti-α1-adrenoreceptor Abs
  - Anti-α2-adrenoreceptor Abs
  - Anti-β1-adrenoreceptor Abs
  - Anti-β1 adrenoreceptor Abs
  - Anti-β1 adrenoreceptor Abs
  - Anti-M1 acetylcholine receptor Abs
  - Anti-M2 acetylcholine receptor Abs
  - Anti-M3 acetylcholine receptor Abs
  - Anti-M4 acetylcholine receptor Abs

Before and after active and sham treatment (before therapy cycle 1/after therapy cycle 1 and before therapy cycle 2/after therapy cycle 2).

## **Study design**

The Mainz immunoadsorption study in adults with post-COVID is a single-center, randomized, controlled, single-blind study with a cross-over design that aims to investigate the potential effect of immunoadsorption therapy on symptom burden in people with post-COVID. The study is being conducted by an interdisciplinary team of investigators.

Included subjects are first randomized to receive either 5 verum treatments (immunoadsorption with TR-350 adsorber) or 5 sham treatments (connection to the Plasauto Sigma without adsorber). All subjects will receive both the real treatment and the sham treatment unless they decide against the respective therapy cycle before the start of the respective therapy cycle. Randomization is only used to determine which treatment is given first.

Participating subjects may withdraw from the study at any time, including after the first therapy cycle or before the second therapy cycle.

The subjects are blinded because they do not know whether the treatment is being carried out with or without an adsorber, and it is also not apparent to the subjects, as the TR-350 adsorber and Plasauto Sigma are set up behind a curtain that is not visible to the patient and remain there throughout the treatment.

The blinding of the subjects serves to assess the actual effect of the therapy without the influence of the therapy arm on the patients' reports. Since the therapeutic effects in the present study are predominantly measured using self-report instruments, such an approach seems essential to minimize the influence of distortions caused by psychological effects of the verum therapy on the study results.


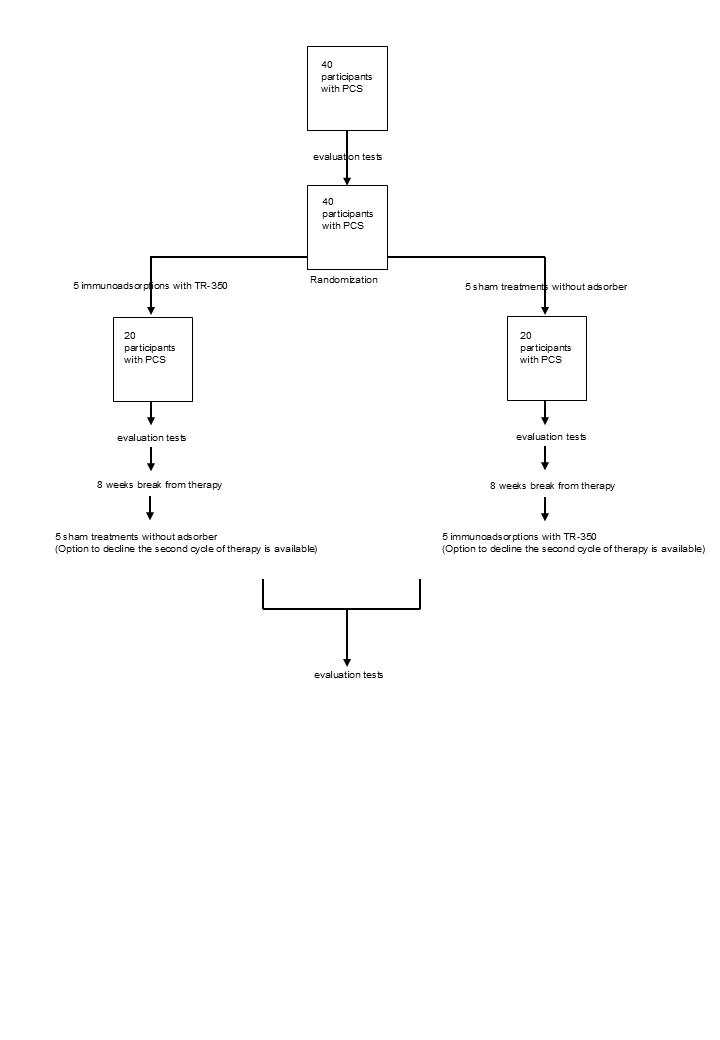


1 Figure Study design and flow chart

## **Study population**

### 8.1 Inclusion criteria:

- Fulfilment of the WHO diagnostic criteria for post-COVID syndrome
- Written consent to participate in the study
- Previous participation in the Gutenberg Post-COVID study
- Minimum age of 18
- PCFS score of at least 2

### 8.2 Exclusion criteria:

- Psychiatric diagnosis
- Allergy to adsorbent materials, materials used in the tube systems, or substances used for immunoadsorption
- Pregnancy
- Medical contraindications to immunoadsorption, such as severe blood clotting disorders or immunodeficiency syndromes
- Existing antibody-mediated autoimmune disease

### 8.3 Recruitment

The subjects will be recruited from the 700 participants in the Gutenberg Post Covid Study. Patients who meet the criteria for post-COVID will be offered participation in the immunoadsorption study in adults with post-COVID.

### 8.4 Number of participants

### A total of 40 subjects are to be included.

## **Study procedure**

### Information and consent

All participants will be informed in a personal meeting, both by means of a written information sheet and in a detailed discussion, about the objectives of the study, the study procedure, the examinations required as part of the study, the active treatment, the sham treatment, and the associated risks and potential benefits of participating in the study. Furthermore, detailed information will be provided about the data collected during the study, how it will be anonymized, and for what purposes the data will be stored. Study participants will also be informed about the preservation of biomaterials obtained during their participation in the study. Any questions the participants may have will be clarified in a personal interview. After a reflection period of at least 24 hours, written consent is then obtained. Consent given once can be revoked at any time during participation in the study without any disadvantages for the participant.

### Collection of data and procurement of biomaterials

Since the test subjects are recruited from participants in the Gutenberg Long COVID Study, a lot of data is already available from surveys conducted as part of that study. When participating in the Gutenberg Long COVID Study, the subjects were already examined by an interdisciplinary team of doctors from preventive cardiology, preventive medicine, and the Neuroimaging Center at Mainz University Medical Center, so that a wide range of laboratory values and examination findings are already available for each participant, which can be used as baseline values. Standardized questionnaires were handed out, personal interviews were conducted, medical physical examinations were performed, and imaging techniques were used. Many of the values required from blood and urine tests were also already determined when participants were included in the Gutenberg Long COVID study.

#### Demographic data

Demographic data is already available from questionnaires and a personal interview conducted as part of the Gutenberg Long Covid Study. The following will be evaluated for the immunoadsorption study:

- Age
- Gender
- Ethnic
- Smoking status
- Highest level of education
- Marital status
- Children
- Current occupation
- Occupation prior to SARS-CoV-2 infection

#### Clinical data

The clinical data is taken from the Gutenberg Long COVID Study, which was collected through questionnaires and personal interviews:

- Pre-existing conditions
- Time of SARS-COV-2 infection
- Vaccination status with regard to SARS-CoV-2
- Symptoms during SARS-COV-2 infection
- Hospitalization status during SARS-COV-2 infection
- Need for oxygen during SARS-CoV-2 infection

#### Laboratory tests and collection of biomaterial

During participation in the study, venous blood samples will be taken at various times by nursing staff or doctors trained in blood sampling. The parameters to be determined serve, on the one hand, to find or rule out alternative causes for the symptoms reported in the context of post-COVID syndrome and, on the other hand, to provide explanations for any improvements or worsening of symptoms during or after therapy. Most of the laboratory values for the individual participants are already available at the time of study inclusion from the examinations carried out during their participation in the Gutenberg Long COVID Study. Some parameters must be determined several times during study participation. Table 2 shows which parameters are determined at which point in the study.

In addition, a urine test is planned several times during the study, for which the subjects must provide at least 50 ml of midstream urine.

All analyses of blood and urine samples are performed in a pseudo-anonymous manner by the Central Laboratory of the University Medical Center Mainz.

Saliva samples for determining cortisol concentrations at different times of the day will be collected during an inpatient stay on the day before the first extracorporeal therapy of the respective cycle, as these must be processed promptly.

| Parameters | Time of collection | Material |
| --- | --- | --- |
| Complete blood count | E, vT1, nT1, vT2, nT2 | Serum |
| Sodium concentration | E, vT1, nT1, vT2, nT2 | Plasma |
| Potassium concentration | E, vT1, nT1, vT2, nT2 | Plasma |
| Calcium concentration | E, vT1, nT1, vT2, nT2 | Plasma |
| Phosphate concentration | E, vT1, nT1, vT2, nT2 | Plasma |
| Transferrin saturation | E, vT1, nT1, vT2, nT2 | Serum |
| Ferritin | E, vT1, nT1, vT2, nT2 | Serum |
| C-reactive protein | E, vT1, nT1, vT2, nT2 | Plasma |
| Antinuclear antibodies | E, vT1, nT1, vT2, nT2 | Serum |
| Daily profile of cortisol concentration in saliva | E, vT1, nT1, vT2, nT2 | Saliva |
| Thyroid-stimulating hormone | E, vT1, nT1, vT2, nT2 | Serum |
| 25-hydroxy vitamin D concentration | E | Serum |
| Vitamin B12 concentration | E | Serum |
| Immunoglobulin G concentration | E, vT1, nT1, vT2, nT2 | Serum |
| Antibodies against α-adrenoreceptors of subtypes 1-2 | E, vT1, nT1, vT2, nT2 | Serum |
| Antibodies against β-adrenergic receptors of subtypes 1-3 | E, vT1, nT1, vT2, nT2 | Serum |
| Antibodies against muscarinic acetylcholine receptors of subtypes M1-M4 | E, vT1, nT1, vT2, nT2 | Serum |
| Venous blood gas analysis | E, T1, T2 | Whole blood |
| Activated partial thromboplastin time (aPTT) | E, vT1, nT1, vT2, nT2 | Citrate plasma |
| International normalized ratio (INR) | E, vT1, nT1, vT2, nT2 | Citrate plasma |
| Fibrinogen concentration | E, vT1, nT1, vT2, nT2 | Citrate plasma |
| Albumin in spontaneous urine | E, vT1, nT1, vT2, nT2 | Midstream urine |
| Creatinine in spontaneous urine | E, vT1, nT1, vT2, nT2 | Midstream urine |
| Total protein in spontaneous urine | E, vT1, nT1, vT2, nT2 | Midstream urine |
| Urine sediment including acanthocyte concentration | E, vT1, nT1, vT2, nT2 | Midstream urine |
| Albumin/creatinine ratio in spontaneous urine | E, vT1, nT1, vT2, nT2 | Midstream urine |
| Total protein/creatinine ratio in spontaneous urine | E, vT1, nT1, vT2, nT2 | Midstream urine |

1 table: Overview of the parameters determined from the respective biomaterial and the time of material collection (E = evaluation tests; T1 = during therapy cycle 1; T2 = during therapy cycle 2; vT1 = before therapy cycle 1; nT1 = after therapy cycle 1; vT2 = before therapy cycle 2; nT2 = after therapy cycle 2).

### Medical-technical examinations

- - - 1. Physical examination, anthropometry, and vital signs

Each study participant undergoes a physical examination by a physician, which includes auscultation of the heart and lungs and a clinical examination of the abdomen. The height and weight of each participant are recorded and used to calculate their body mass index.

Vital signs are recorded by medically trained personnel using electronic devices. Resting blood pressure is determined oscillometrically using an upper arm cuff after 5 minutes of physical rest in a sitting position, heart rate is determined using pulse oximetry, body temperature is measured via tympanic measurement with a laser thermometer, and respiratory rate is counted.

The results of the physical examination, anthropometry, and vital signs are taken from the data of the Gutenberg Long COVID Study.

In addition to the measurements of vital signs, which are determined for the collection of study data, heart rate, oxygen saturation, and blood pressure are continuously monitored during each therapy session, both during the active and placebo therapy, or every ten minutes in the case of blood pressure, using electrical devices.

- - - 1. 12-channel ECG

A 12-channel ECG is recorded for each study participant as part of the Gutenberg Long COVID Study. Data from the findings of these 12-channel ECGs will be transferred to the immunoadsorption study, as this could provide clues to the causes of the symptoms experienced by the participants. The rhythm, QT time, ST segment findings, and any block patterns are transferred.

- - - 1. Spirometry

In order to record the lung function of the subjects and to determine the possible influence of the therapy cycles on lung function, as well as to evaluate a possible correlation between the reported dyspnea symptoms and the measured lung function, spirometry will be performed on each participant. Initially, each subject has already undergone spirometry, which was performed during participation in the Gutenberg Long COVID Study. After each therapy cycle, another spirometry test will be performed by the Center for Preventive Cardiology. Parameters for determining obstructive and restrictive ventilation disorders will be measured, as well as the diffusion capacity of the lungs.

- - - 1. Neurological examinations

To objectively assess neurological symptoms, neurological examinations will be performed on each study participant. The initial examination will take place when participants enroll in the Gutenberg Long COVID study. To determine the possible effects of the therapy on neurological symptoms, identical examinations will also be performed after each therapy cycle.

*Vibration sensitivity*

The vibration perception threshold is measured using a tuning fork on the metatarsophalangeal joint of the big toe as an indication of a possible sensory disorder.

*Force measurement*

Muscle strength is determined, for example, by measuring the pressure exerted by the hand muscles when squeezing a rubber balloon with the dominant hand. The force applied is measured using a vigorimeter.

*Smell test*

The smell test is performed using a test battery of standardized smell sticks containing eight different smells.

*Testing pain sensitivity*

Pain sensitivity is tested using a pinprick stimulator and the sensation of pressure pain is measured using a Wagner pressure pain algometer.

- - - 1. Neurocognitive testing

Various neurocognitive functions are tested using the Montreal Cognitive Assessment (MoCA). This is a ten-minute test with various tasks that can be scored with a total of up to 30 points. It tests abilities in the areas of memory, attention, verbal abstraction, visuospatial abilities, concentration, and language comprehension.

- - - 1. Post-COVID-19 Functional Status Scale (PCFS)

The PCFS is a self-assessment tool that helps to objectively measure and compare the severity of symptoms experienced by people with post-COVID. Patients are asked to describe their condition or fill out a questionnaire. Each condition is assigned a value. The greater the severity of the symptoms described by the condition, the higher the point value on a scale of 0-4. The PCFS has been validated on cohorts of post-COVID patients and is therefore particularly suitable for assessing symptom severity.

- - - 1. Assessment of the severity of fatigue symptoms

Three different questionnaires are used to objectively assess fatigue symptoms and the resulting limitations in everyday life. These are the Chalder Fatigue Scale, the Bell Score, and the Multidimensional Fatigue Inventory (MFI-20).

- - - - 1. *Chalder Fatigue Scale*

The Chalder Fatigue Scale is a set of 11 questions that assesses two dimensions of fatigue symptoms: physical performance and mental performance. Each question is answered using a 4-point Likert scale. It takes an average of 2-3 minutes to complete and can be repeated every 4 weeks. In the validation study, a sensitivity of 75.5% for chronic fatigue syndrome and a specificity of 74.5% were achieved.

The Chalder Fatigue Scale should be completed by each study participant as part of the evaluation examinations, before and after therapy cycle 1, before and after therapy cycle 2, and in the follow-up examination. It is completed digitally using a questionnaire on a tablet (iPad).

- - - - 1. *Bell Score*

The Bell Score is an assessment tool that can be used by both the patient and the examiner to measure the degree of limitation in daily life caused by fatigue symptoms. The limitation can be rated on a scale of 11, from 0 (bedridden) to 100 (no limitations, normal ability to perform all activities of daily living).

The Bell Score should be completed by each study participant as part of the evaluation examinations before and after therapy cycle 1, before and after therapy cycle 2, and in the follow-up examination. It is completed digitally using a questionnaire on a tablet (iPad).

- - - 1. Medical Research Council (MRC) Dyspnea Scale

In order to objectify the perceived dyspnea symptoms, make them more comparable, and be able to assess the possible effect of therapy on dyspnea symptoms, patients are asked about their symptoms using the MRC Dyspnea Scale, which uses 4 statements assigned a point value of 1-4 that describe situations in which dyspnea occurs. The subjects must then select the statement that best describes their situation.

- - - 1. Cranial MRI

All subjects are undergoing a cranial MRI at the Mainz Neuroimaging Institute as part of the Gutenberg Long COVID Study. The results will be used in the study " " to assess whether the patients' symptoms could have causes other than long COVID and whether patients with certain abnormalities respond particularly well or particularly poorly to therapy, as well as to investigate a possible correlation between the presence of autoantibodies and abnormalities in the MRI. T1, T2-TSE FLAIR, and rsfMRI will be measured on a Siemens Magnetron TRIO T3.

| Examination | Results from the Gutenberg Long Covid Study (evaluation) | Time |
| --- | --- | --- |
| Medical history interview and questionnaire | Yes | E |
| Anthropometry | Yes | E |
| Vital signs (HR, RR, temp., respiratory rate) | no | E, T1, T2 |
| 12-channel ECG | Yes | E |
| Spirometry | Yes | E, nT1, vT2, nT2, F |
| Neurological examination | Yes | E, nT1, vT2, nT2, F |
| Neurocognitive testing | Yes | E, nT1, vT2, nT2, F |
| PCFS score | No | E, nT1, vT2, nT2, F |
| Chalder fatigue scale | no | E, nT1, vT2, nT2, F |
| Bell score | No | E, nT1, vT2, nT2, F |
| MRC score | no | E, nT1, vT2, nT2, F |
| Blood sampling | yes | E, T1, nT1, T2, vT2, nT2, F |
| Urine analysis | Yes | E, nT1, vT2, nT2, F |
| Cranial MRI | yes | E |
| Psychological questionnaires | yes | E, nT1, vT2, nT2, F |

Table2 : Overview of examinations performed, proportion of examinations whose results can be used from the Gutenberg Long COVID study as part of the evaluation examinations, and when the respective examinations are performed. (E = evaluation examinations; T1 = during therapy cycle 1; T2 = during therapy cycle 2; vT1 = before therapy cycle 1; nT1 = after therapy cycle 1; vT2 = before therapy cycle 2; nT2 = after therapy cycle 2, F = follow-up; for most examinations, the evaluation examination is equivalent to the examination before therapy cycle 1).

- 1. Informing participants about examination results

After each examination, the test subjects will receive brief information if there are any relevant examination results. After completion of the study, participants will receive a detailed written report of the findings and an appointment will be offered to discuss the findings.

9.4 Timeline for individual study participants


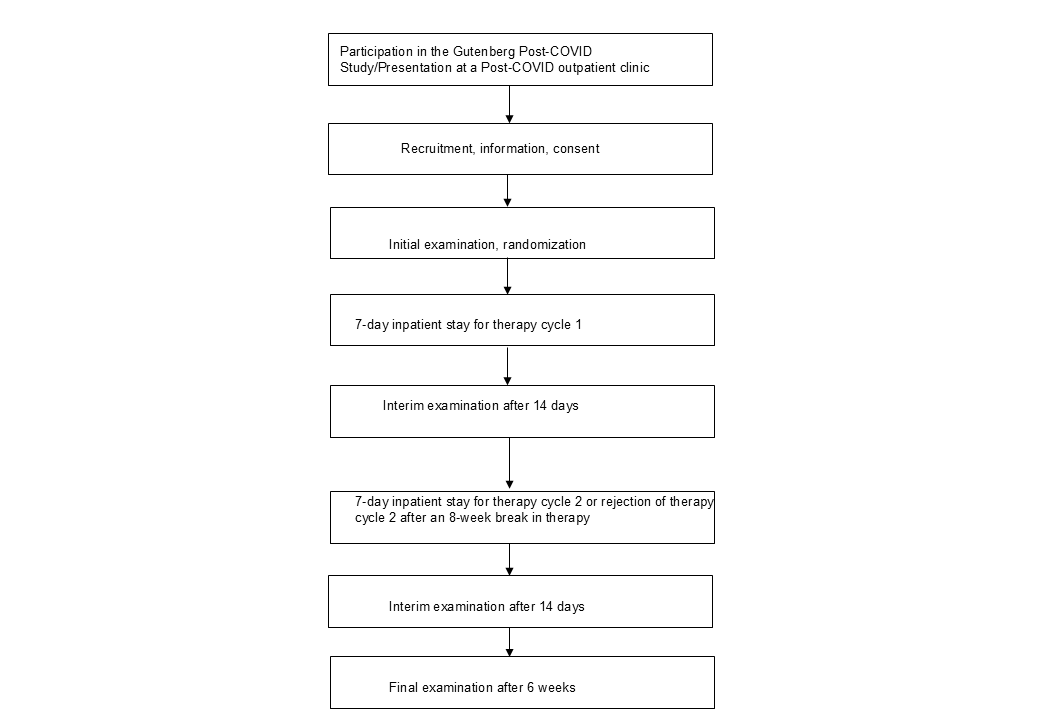


2 : Flowchart of the schedule for individual study participants

| Examination | Duration |
| --- | --- |
| Neurocognitive testing | 15 |
| PCFS score, Chalder Fatigue Scale, Bell Score, MFI-20 | 10 minutes |
| MRC score | 5 |
| Blood sample | 5 |
| Measurement of vital signs | 10 |

Table3 : Duration of examinations in the evaluation examination block

| Examination | Duration |
| --- | --- |
| Inpatient stay | 7 days, including the following measures |
| Neurocognitive testing | 15 minutes |
| PCFS score, Chalder Fatigue Scale, Bell Score, MFI-20 | 10 |
| MRC score | 5 |
| Blood sample | 5 |
| Measurement of vital signs | 10 |
| Neurological examination | 15 |
| Spirometry | 10 |
| If venous cannulation is not possible: Shaldon device | 15 |
| Venous cannulation | 5 |
| Extracorporeal therapy 1 | 240 |
| Venous cannulation | 5 |
| Extracorporeal therapy 2 | 240 |
| Venous cannulation | 5 |
| Extracorporeal therapy 3 | 240 |
| Rest day |  |
| Extracorporeal therapy 4 | 240 |
| Day off |  |
| Extracorporeal therapy | 240 |

Table4 : Duration of examinations and interventions in the block "7-day inpatient stay for therapy cycle 1" and "9-day inpatient stay for therapy cycle 2"; blood samples indicated above as being taken before or after therapy are taken during extracorporeal therapy to avoid additional punctures.

| Examination | Duration |
| --- | --- |
| Medical history interview | 30 |
| Neurocognitive testing | 15 |
| PCFS score | 10 minutes |
| MRC score | 5 |
| Blood sampling | 5 |
| Measurement of vital signs | 10 |
| Neurological examination | 15 |
| Spirometry | 10 |

Table5 : Duration of examinations in the "Follow-up visit 4 weeks" block

## 9.5 Administration of treatment

All study participants receive 5 blinded extracorporeal therapies with the Plasauto Sigma from Diamed with a TR-350 adsorber inserted and 5 extracorporeal therapies with the same device with no adsorbers inserted. The order, i.e., whether the verum treatment (adsorber inserted) or the sham treatment with no adsorbents is performed first, will be assigned randomly. Immunoadsorption and sham treatment are performed, depending on the condition of the veins, via two large-lumen peripheral venous cannulas or, if this is not possible due to poor vein condition, via a Shaldon catheter, which is inserted into the right or left internal jugular vein under ultrasound guidance after the patient has been informed of the risks and has given their consent. Anticoagulation is performed during both sham treatment and actual immunoadsorption using heparin (bolus of 2000iE, then flow rate of 2000iE/h) and the PA-420 tube system is used. The blood flow is set to 60 ml/min and the plasma flow to 20ml/min for both therapies. The exchange volume depends on the patient's size and body weight and is 2-2.5 L per session.

## 9.6 Total duration of the study

The total duration of the study is set at 24 months. This study duration is based on the fact that 40 participants are to be recruited and then treated. One treatment cycle lasts one week. Each participant receives two treatment cycles, so that two weeks of pure therapy must be calculated per participant. Since the capacity for extracorporeal procedures at the study center is limited, this long period of time is necessary to be able to carry out a sufficient number of therapies. Twenty months are therefore scheduled for the therapy, two months for the necessary preliminary examinations and study inclusion, and two months for the follow-up visits.

## **Benefit-risk assessment**

10.1 Individual benefits associated with participation in the study

- Detailed examination and objectification of the symptoms and severity of post-COVID syndrome
- Investigation of alternative causes for the symptoms and associated findings about the individual's state of health
- Advice on further treatment options in the event of a diagnosis of a disease within the scope of the study examination
- Prospect of symptom relief if the investigated immunoadsorption is effective in post-COVID

10.2 Burdens and risks associated with participation in the study

- Pain, hematomas, accidental arterial puncture, damage to peripheral nerves during peripheral venous cannulation before each immunoadsorption or sham adsorption, reduction of risk through ultrasound-guided cannulation
- Insertion of a central venous dialysis catheter (Shaldon catheter) if peripheral venous cannulation is not possible, with risk of injury to the carotid artery, bloodstream infections, risk of lung injury with development of pneumothorax Reduction of risks through sterile working practices, ultrasound-guided catheter placement, and the extensive experience of the physicians involved in the study in catheter placement
- Allergic reaction to heparin, adsorbent material, or tubing materials
- Development of heparin-induced thrombocytopenia (HIT) with the development of thromboembolism, a drop in platelet concentration, and the development of bleeding
- Stress caused by two hospital stays of 7 days each

10.3 Discontinuation criteria

- Participation in the study is voluntary. Patients may withdraw from the study at any time without giving a reason and without any subsequent disadvantage.
- Definition of adverse events (AE): Adverse events are defined as any disturbances in well-being observed during the course of treatment.
- Serious adverse event (SAE): An SAE is life-threatening or fatal. It leads to significant or persistent disability, hospitalization, and includes any significant risk that may harm the patient and is related to the treatment administered.
- Non-serious adverse event (AE): All events that do not fall under the SAE category.

10.4 Statement on medical defensibility

Peripheral venous cannulation, insertion of central venous dialysis catheters, and the use of heparin as an anticoagulant in extracorporeal therapies are standard procedures in extracorporeal therapies and are performed several times a day in the dialysis department of the University Medical Center's nephrology unit. Immunoadsorption is also performed regularly, and there is extensive experience with this therapeutic procedure. All measures are carried out in accordance with the 2019 therapeutic apheresis standards of the German Society of Nephrology.

## **Biometrics**

In a published post-COVID cohort from Germany (Lier et al., Frontiers Neurol 2022, DOI: 10.3389/fneur.2022.988359), the median MoCA score was 27 with a interquartile range of 25 to 28. The null hypothesis is that immunoadsorption cannot reduce the score by 2 points, i.e., below the MoCA threshold. To achieve a power of 90%, according to a calculation using the G*Power Version 3.1.9.7 utility program (Faul et al., Behav Res Methods 2009), two treatment groups of 19 subjects each are required (effect size 1.0, one-sided Wilcoxon Mann-Whitney test with alpha level 0.05 and 1:1 randomization). To account for possible dropouts, the number of cases is set at 2x 20 subjects.

1. **Data management and data protection**

- Laboratory parameters will be collected. In addition, patients will complete questionnaires that allow conclusions to be drawn about their quality of life, symptom burden, cognitive abilities, and resilience in everyday life.
- Medical examinations will be carried out to assess lung function, strength, central and peripheral nervous system function, heart rhythm, and vital signs.
- Patient data and other information about blood samples and the findings of the above-mentioned examinations will be archived in a pseudonymized, password-protected database.
- The data and samples can therefore only be traced back using a key that is accessible only to the study director and the medical director of the First Medical Clinic. The data is labeled with the same key as the samples. The requirements of medical confidentiality and data protection are therefore ensured within the framework of this study.
- The data will not be passed on to third parties. The data will be stored for 10 years.
  - The data will be stored in pseudonymized form.
  - Consent can be revoked at any time in writing or verbally without giving reasons.

1. **Handling of biomaterials**

- Patient data and other information about the blood samples and the examination findings will be archived in pseudonymized form in a computerized database.
- The data and samples can therefore only be traced back using a key that is accessible only to the study director and the medical director of the First Medical Clinic. The data is labeled with the same key as the samples. The requirements of medical confidentiality and data protection are therefore maintained within the framework of this study. The database was evaluated using queries in Structured Query Language (SQL). The analyses were performed using the statistical program SPSS for Windows Version 22.

1. **Subject insurance, accident insurance**

Test subject insurance was taken out with the HDI insurance company for damages up to €500,000 per test subject and a total damage amount of €5,000,000.

**15. Publication rules**

The results will be published at national and international conferences and in specialist journals with an editorial board and review process.

**Consent of the head of the institution**

The director of the First Medical Clinic and Polyclinic at the University Medical Center Mainz, Univ-Prof. Dr. med. Peter R. Galle, agrees to the implementation of the project.

_________________ ___________________________________________

###### Place, date Univ.-Prof. Dr. med. Peter R. Galle

I. Department of Medicine, University Medical Center of Johannes Gutenberg University Mainz

Signatures of the study director

_________________ ____________________________________________

Place, date Univ. Prof. Julia Weinmann-Menke

I. Department of Medicine, University Medical Center of Johannes Gutenberg University Mainz

**Ammendments to the Mainz immune adsorption study in adults with post-COVID (IAMPOCO)**

1. Study participants do not necessarily have to have participated in the Gutenberg Post-COVID Study prior to participating in the study, but may also be included if comparable examinations and assessments have been carried out by post-COVID outpatient clinics or post-COVID specialist practices.

2. The only neurological examinations carried out as part of the study are pain assessment using a numerical analog scale and cognitive assessment using the MocA.

3. Anticoagulation for both immune adsorption and sham treatment is achieved using citrate anticoagulation and continuous heparin administration at 1000iE/h.
